# Epigenetic Mechanisms Histone Deacetylase–Dependent Regulate the Glioblastoma Angiogenic Matrisome and Disrupt Endothelial Cell Behavior *In Vitro*

**DOI:** 10.1016/j.mcpro.2024.100722

**Published:** 2024-01-23

**Authors:** Aline Menezes, Glaucia Julião, Fernanda Mariath, Ana Luiza Ferreira, Maria Cecilia Oliveira-Nunes, Lara Gallucci, Joseph Albert Medeiros Evaristo, Fábio César Sousa Nogueira, Denise de Abreu Pereira, Katia Carneiro

**Affiliations:** 1 Instituto de Ciências Biomédicas e Programa de Pós-graduação em Medicina (Anatomia Patológica), UFRJ/RJ, Rio de Janeiro, Rio de Janeiro, Brazil; 2 Laboratório de Estudos Avançados em Jornalismo, UNICAMP/SP, São Paulo, São Paulo, Brazil; 3 Carisma Therapeutics, Philadelphia, Pennsylvania, USA; 4 Laboratory of Proteomics, LADETEC, Institute of Chemistry, Federal University of Rio de Janeiro, Rio de Janeiro, Brazil; 5 Proteomics Unit, Institute of Chemistry, Federal University of Rio de Janeiro, Rio de Janeiro, Brazil; 6 Programa de Oncobiologia Celular e Molecular, Coordenação de Pesquisa, Instituto Nacional do Câncer- INCA/RJ, Rio de Janeiro, Rio de Janeiro, Brazil

**Keywords:** Epigenetics, histone Deacetylase, Glioblastoma, secretome, matrisome

## Abstract

Glioblastoma (GBM) is the most aggressive brain tumor and different efforts have been employed in the search for new drugs and therapeutic protocols for GBM. Epitranscriptomics has shed light on new druggable Epigenetic therapies specifically designed to modulate GBM biology and behavior such as Histone Deacetylase inhibitors (iHDAC). Although the effects of iHDAC on GBM have been largely explored, there is a lack of information on the underlaying mechanisms HDAC-dependent that modulate the repertoire of GBM secreted molecules focusing on the set of Extracellular Matrix (ECM) associated proteins, the Matrisome, that may impact the surrounding tumor microenvironment. To acquire a better comprehension of the impacts of HDAC activity on the GBM Matrisome, we studied the alterations on the Matrisome-associated ECM regulators, Core Matrisome ECM glycoproteins, ECM-affiliated proteins and Proteoglycans upon HDAC inhibition *in vitro* as well as their relationship with glioma pathophysiological/clinical features and angiogenesis. For this, U87MG GBM cells were treated for with iHDAC or vehicle (control) and the whole secretome was processed by Mass Spectrometry NANOLC-MS/MS. *In silico* analyses revealed that proteins associated to the Angiogenic Matrisome (AngioMatrix), including Decorin, ADAM10, ADAM12 and ADAM15 were differentially regulated in iHDAC *versus* control secretome. Interestingly, genes coding for the Matrisome proteins differentially regulated were found mutated in patients and were correlated to glioma pathophysiological/clinical features. *In vitro* functional assays, using HBMEC endothelial cells exposed to the secretome of control or iHDAC treated GBM cells, coupled to 2D and 3D GBM cell culture system, showed impaired migratory capacity of endothelial cells and disrupted tubulogenesis in a Fibronectin and VEGF independent fashion. Collectively, our study provides understanding of epigenetic mechanisms HDAC-dependent to key Matrisomal proteins that may contribute to identify new druggable Epigenetic therapies or gliomagenesis biomarkers with relevant implications to improve therapeutic protocols for this malignancy.

## Highlights

• HDAC activity modulates the repertoire of GBM-secreted molecules.
• HDAC activity inhibition alters the GBM angiogenic Matrisome, the AngioMatrix.
• The secretome of GBM cells impairs endothelial cells migration and tubulogenesis.

## Graphical Abstract

**Figure:**
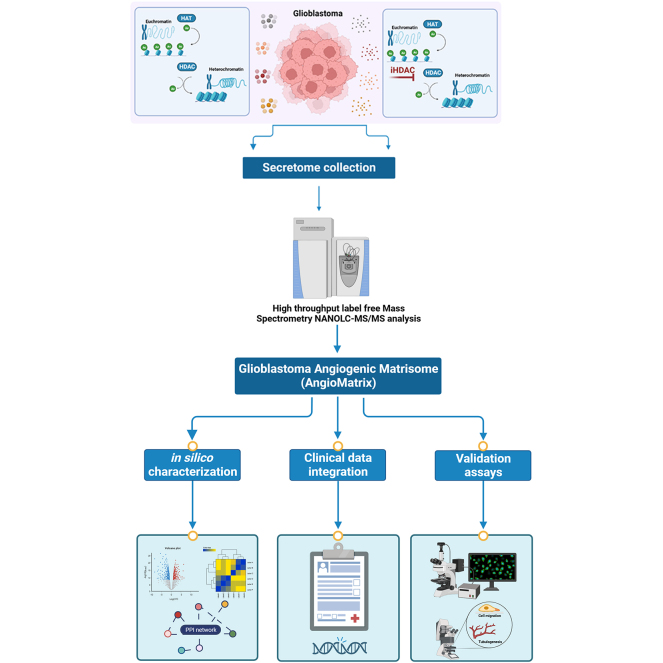

## In Brief

Glioblastoma (GBM) is the most aggressive brain tumor. Histone deacetylase inhibitors (iHDAC) have emerged as druggable epigenetic therapies for GBM. The effects of iHDAC on the GBM matrisome is currently elusive. In this study, we performed a high throughput proteomic analysis of the GBM matrisome upon pharmacological knockdown of HDAC activity in GBM cells. We characterize the GBM angiogenic matrisome signature, the AngioMatrix, and its impact on endothelial cell behavior, strengthening our comprehension of GBM biology.

Glioblastoma (GBM) is the most prevalent primary malignant brain tumor in adults and remains almost invariably lethal due to its aggressive and invasive behavior (1). Despite many clinical trials have been put forward, the standard treatment consists of surgical resection, followed by temozolomide (TMZ) chemotherapy and radiation treatment (2, 3). Therefore, due to the low prognosis, different efforts have been employed in the search for new drugs and therapeutic protocols for this malignancy (4). Due to the accelerated gain of knowledge on GBM genomic, transcriptomic and proteomic, a better comprehension of core molecular signatures linking tumor transcriptional states, or epitranscriptomics, and clinical/pathophysiological features have improved the characterization of new druggable Epigenetic therapies specifically designed to modulate GBM biology and behavior. Indeed, a range of clinical trials have been focusing on molecular targets previously described as classical markers of GBM epigenetic signatures, such as O^6^-methylguanine–DNA methyltransferase (MGMT) gene methylation and histone posttranslational modifications (PTM), as putative epigenetic biomarkers for tumor diagnosis and sub-type classification, prognosis as well as for personalized therapeutical targets as an option for eligible patients (5).

Because histone PTMs patterns are frequently disrupted in a wide variety of cancers, the enzymes critically involved in the dynamic balance of histone PTMs have received increased attention as druggable targets for oncologic research (6, 7). A class of drugs that has emerged as a promising candidate for GBM is Histone Deacetylase inhibitors (iHDAC). In fact, preclinical studies have demonstrated the efficacy and security of different iHDACs as antitumor agents for GBM therapy, especially when associated with other therapies, including chemotherapy and radiation (8, 9, 10, 11). The use of iHDACs in clinical trials has received great support from the literature, that has been characterizing the biological effects of this class of drugs, including the modulation of genes involved in DNA repair, formation of mitotic spindles and homologous chromosome segregation, ultimately leading to glioma apoptosis *in vitro* (12, 13, 14). In agreement, a high activity of HDAC has also been associated with tumor progression and migration, corroborating the pharmacological inhibition of HDACs as an interesting anti-cancer target (15).

Due to the increasing interest in translating iHDACs from the bench to the oncologic clinic, it is imperative to improve our comprehension on the underlying mechanisms that this class of drugs modulate on the tumor microenvironment, such as angiogenesis. For this, the coupling of epigenetic and biochemical profiling techniques could shed light on the interplay between specific epigenetic states and functional molecular signatures that might shape the extracellular microenvironment. In this work we have focused on the putative role of iHDACs in modulating the repertoire of GBM secreted molecules, which in turn, may directly modulate the behavior of endothelial cells. Protein secretion is involved in several cellular mechanisms, such as cellular homeostasis, cell-cell communication, and metastatic niche preparation (16). The secreted proteins will play a critical role in intercellular communication and will be in charge with the interactions between the tumor cells and the tumor microenvironment. These proteins in turn will mediate tumor growth, proliferation, invasion and angiogenesis into the adjacent tissue (16). To better investigate the GBM secretome upon iHDAC treatment *in vitro*, we employed a high throughput label free methodology of protein identification and quantification based on mass spectrometry followed by *in silico* studies using Reactome pathway enrichment analyses, STRING Protein-protein interaction (PPI) as well as *in vitro* assays. The *in silico* study revealed that HDAC inhibition was correlated with a disrupted pattern in the abundance of a limited number of secreted proteins that are mostly Matrisome-associated ECM regulators, Core Matrisome ECM glycoproteins, ECM-affiliated proteins and Proteoglycans. ECM-associated molecules with critical implications on angiogenic Matrisome, the AngioMatrix, such as Decorin, ADAM10, ADAM12 and ADAM15 were also observed in the absence of significative changes in the abundance of proteins classically involved in ECM organization, such as Fibronectin and Integrins, and angiogenesis, such as vascular endothelial growth factor (VEGF). Furthermore, *in vitro* functional assays using HBMEC endothelial cells, coupled to 2D and 3D GBM cells culture system, confirmed that the secretome obtained from iHDAC treated GBM cells drastically disrupted endothelial cells behavior, impairing migratory capacity of endothelial cells coupled to a deficient tubulogenesis in a Fibronectin and VEGF independent fashion. Taken together our results describe for the first time in the literature the relevance of epigenetic mechanisms HDAC-dependent for the molecular signature of the GBM secretome focusing on the Matrisome and its components involved in angiogenesis shedding light on putative molecular targets to approach this malignancy.

## Experimental Procedures

### Proteomic Analysis

#### Preparation of Conditioned Medium – Secretome

U87MG GBM cells were obtained from Banco de Células do Rio de Janeiro, BCRJ. These cells were seeded at a density of 7 × 10^5^ cells on 150 cm^2^ bottles (CORNING) with Dulbecco's Modified Eagle Medium (DMEM, low glucose; Sigma-Aldrich) supplemented with 10% Fetal Bovine Serum (FBS), (LGC Biotechnology), and 0.1 mg/ml penicillin/streptomycin (PS) until reaching a confluence of 90%. Cultures were maintained at 37 °C and with 5% CO_2_. Once cells were confluent, cultures were washed once with Phosphate Buffer Saline (PBS) supplemented with 2M Sodium Chloride (NaCl_2_), followed by two washes with PBS, to remove excess FBS and salts contained in the culture medium. Cells were then kept on DMEM without phenol red (Sigma-Aldrich) and without FBS. The group under HDAC activity inhibition (here referred as iHDAC), received media supplemented with iHDACs Trichostatin A (TSA, T1952 Sigma-Aldrich), at a concentration of 100 nM. For the control group, the vehicle Dimethylsulfoxide (DMSO, Sigma-Aldrich) was used. Treatments were performed for 72 h. Data shown consists of two biological replicates of each group. iHDACs concentration were chosen based on previous characterization of our group (15).

#### Dialysis of the Conditioned Medium - Secretome

After 72 h of treatment, the conditioned media, here referred as Secretome, was collected and spun down to remove cells and debris. The media was concentrated by centrifugation at 5000 rpm for 30 min, in AMICON ULTRA 4 3KD- 50 ml tubes (Merck Millipore). The final volume of recovered media was 250 μl per sample. Protease inhibitor Halt (Halt Protease Inhibitor Cocktail, Thermo Fisher) was added, and protein quantification was performed using the Qubit Protein Assay Kit method (Molecular Probes), according to the manufacturer’s instructions.

#### Protein Digestion and Peptide Purification

After protein quantification, we performed in solution protein digestion protocol using trypsin. A 100 μg of total protein were diluted in 20 μl of 7M Urea and 2M Thiourea in 0.2 M HEPES (4-(2-hydroxyethyl)-1-piperazineethanesulfonic acid). Then Dithiothreitol solution (DTT) was added at a 10 mM final concentration, followed by incubation for 1 h at 30 °C in the Thermomixer (Eppendorf) without stirring. After incubation, Iodoacetamide solution (IAA) was added at a final concentration of 40 mM and the solution was incubated for 30 min at room temperature and protected from light. The solution obtained was diluted at 10 times with standard LC-MS deionized water and trypsin solution (Sequencing Grade Modified Trypsin-PROMEGA V511A) to a final concentration of 1:50 trypsin/protein (w/w), being then incubated overnight at 37 °C in the Thermomixer under 900 rpm rotation. After that, the samples were resuspended in 1% formic acid and passed through C18 microcolumns (ZipTip) for desalination of the peptides. The eluted portion of the supernatant was transferred to injection vials, which were analyzed by NANOLC-MS/MS.

#### Analysis by NANOLC-MS/MS

The peptides were solubilized in 0.1% formic acid and analyzed using an Easy1000 nanoLC system (Thermo Fisher) coupled to the Quadrupole Orbitrap mass spectrometer (Q Exactive Plus, Thermo Scientific). For each sample, a volume of 4 μl (1 μg of peptides), was applied to a Trap column with 200 μm internal diameter and 2 cm long packed with Reprosil-Pur C18 resin (Dr Maisch GmbH HPLC), with pores of 200 Å and 5 μm particle size (packaged in the laboratory). The peptides were eluted in an analytical column with 75 μm in diameter and 25 cm in length packed with Reprosil-Gold C18 resin (Dr Maisch GmbH HPLC), with pores of 300 Å of 3 μm granulometry (packed in the laboratory). Peptide separation was performed using a gradient of 98% solvent A (ACN 5% and formic acid 0.1%) to 20% of solvent B (ACN 95% and 0.1 formic acid) for 85 min, 20 to 40% solvent B in 22 min and 40 to 95% solvent B in 5 min. After the column was rebalanced with solvent A. The Orbitrap mass spectrometer was controlled by the Xcalibur 2.2 software, which was programmed to operate by the data dependent acquisition (DDA) mode. The spectrum of MS1 was acquired with a resolution of 70,000 to 200 m/z (mass/charge). The reading of the MS1 spectrum was performed using 10e6 ions (AGC) and 50 ms of Maximum IT. The reading spectrum comprised ions with 375 to 2000 m/z.The 20 most intense ions were fragmented and then subjected to MS2 acquisition, using induced collision dissociation (HCD) and a range of 200-2000 m/z. MS2 resolution was 17,500 to 200 m/z, AGC of 10e5 ions, Maximum IT of 100 ms. 1.2 m/z ion isolation window, normalized collision energy (NCE) of 30, dynamic exclusion time was 60s. Peptides with undetermined charges and +1 were rejected. The fractions were injected three times, that is, they made up three technical replicates of each of the two biological samples. To check the reproducibility of the replicates we use The Pearson correlation between the three technical runs of each sample (Supplemental Fig. S1). Then we filtered the proteins identified between the two biological replicates of TSA1 and TSA2 (917 proteins) and the two biological replicates of DMSO1 and DMSO2 (808 proteins), that were used to compare the samples (Supplemental Fig. S2).

#### Data Analysis

Raw files were processed by the Proteome Discoverer (PD) 2.1 software (Thermo Scientific) and spectral data were searched using Sequest HT-Percolator Validator algorithm. The UniProt database, limited to the *Homo sapiens* reference proteome set was downloaded from the UniProt consortium in June 2017 and merged with the Global proteome machine (GPM) common contaminants resulting in 42,227 proteins entries (17). The parameters used in PD Sequest HT node were full-tryptic search space, up to two missed cleavages allowed for trypsin, precursor mass tolerance of 10 ppm, and fragment mass tolerance of 0.05 Da. Carbamidomethylation of cysteine was included as fixed modification, and methionine oxidation and protein N-terminal acetylation were included as dynamic modifications. To estimate the False Discovery Rate (FDR) of <1% and protein inference we used the node Percolator present in the Proteome Discovery 2.1 (PD2.1) using maximum parsimony. A cutoff score was established to accept a false-discovery rate (FDR) of 1% at the protein and peptide level. Protein quantification was done using the Precursor Ions Area Detector node, where the average of up to the 3 most intense peptides was used for protein quantification, being considered unique peptides. The mass spectrometry proteomics data have been deposited to the ProteomeXchange Consortium *via* the PRIDE (18) partner repository with the dataset identifier PXD022982 at PRIDE (https://www.ebi.ac.uk/pride/) username: reviewer_pxd022982@ebi.ac.uk password: OBpIGRa2.

#### *In silico* Analysis

Proteins distributions were done with the platform InteractVenn diagram (19). The signaling pathways enrichment analysis was done with Reactome Knowledgebase (https://reactome.org) version 85 released on June 6th, 2023 (20). Using statistical Overrepresentation *p*-value Highest confidence (score 0.900) protein-protein interactions and relationship between proteins were investigated *via* the STRING database (21) of protein-protein interactions using the accession numbers of all differentially expressed proteins. To classify the proteins belonging to the Extracellular Matrix, we used the Matrisome Classification system (Matrisome AnalyzeR), a suite of tools including a web-based application (https://sites.google.com/uic.edu/matrisome/tools/matrisome-analyzer) and an R package (https://github.com/Matrisome/MatrisomeAnalyzeR) (22). To analyze the statistical significance of protein quantifications in volcano plot, we used Perseus version 1.6.10.50 software (23). The abundance of the proteins was analyzed using Heatmap with the Morpheus software (https://software.broadinstitute.org/morpheus). To evaluate the survival curves and transcriptional subtypes we used the cBioPortal (24, 25). For mutation analysis we have used the Catalog of Somatic mutation in cancer (COSMIC at https://cancer.sanger.ac.uk/cosmic) and Functional Analysis through Hidden Markov Models (http://fathmm.biocompute.org.uk/).

### Validation of Differentially Expressed Proteins

#### Cell Culture

U87MG GBM cell line was genotyped using microsatellite markers of human lineages, at the Laboratório de Metabolismo Macromolecular Firmino Torres de Castro (Instituto de Biofísica Carlos Chagas Filho, UFRJ). The cells were cultured with DMEM supplemented with 10% FBS, plus 0.1 mg/ml PS (Penicillin and Streptomycin) and maintained at 37 ° C and with 5% CO2. For 2D experiments, cells were seeded on coverslips in 24 well-plates, at a density of 2.5 × 10^4^ cell/well for 24 h before starting the treatment described below. For the generation of U87MG oncospheres (3D), non-adherent petri dishes (60mm-Prolab) were used to seed 10^6^ cells/dish with a volume of 1 ml of culture medium. The plate was kept tilted and the culture medium kept in the smallest possible area without disturbance. The oncospheres were treated immediately, for 72 h as described below (26).

#### Drug Treatment

Cells from the iHDAC group were treated with 100 nM Trichostatin (TSA, T8552 Sigma). The control group was treated with the vehicle, DMSO. All treatments were carried for 72 h. Drug concentrations were standardized according to previous work published by our group (15).

#### Cell Cycle Analysis – Flow Cytometry

U87MG cells were treated as described above, and after 72 h in culture, cells were harvested by trypsinization and cell cycle and cell death analysis were performed by quantitation of DNA content according to Vindelov’s protocol (26) Briefly, U87-MG cells were resuspended in 400 *μ*l propidium iodide solution (PBS, 0.1% Triton X-100, 0.1% RNAse, and 50 *μ*g/ml propidium iodide) and incubated on ice for 15 min. Subsequently, cells were analyzed by flow cytometry, using a FACSCanto (BD Biosciences) operated by FACSDiva software, and at least 20,000 events were collected per sample. Cell doublets were gated out using FSC-A *versus* FSC-H profiles. All data were analyzed using FACSDiva software (Version 8.0.1)

#### Immunocytochemistry

Upon treatment the cells were immunostained for the following Extracellular matrix protein Fibronectin using the antibody obtained from “The Developmental Studies Hybridoma Bank, created by the NICHD of the NIH and maintained at The University of Iowa, Department of Biology, Iowa City, IA 52242”: 1- Fibronectin, cell binding domain - P1H11 was deposited to the DSHB by Wayner, E.A. (DSHB Hybridoma Product P1H11). For this assay, the cells were fixed in 4% paraformaldehyde in PBS for 5 min. Permeabilization was performed with 0.3% Triton X-100 in PBS, followed by blockage with 5% BSA (Bovine Serum Albumin; Sigma Aldrich) in PBS for 1 h at room temperature. After blocking, washes were performed using 1x PBS for 3 times/10 min followed by incubation with the primary antibody mentioned above at a concentration of 0.3 μg/ml overnight. Upon PBS washes cells were incubated with secondary antibody, anti-mouse Alexa 488 (1:400, Molecular Probes), followed by actin staining with Phalloidin 546 (1:400, Molecular Probes) and DAPI (1:400), for 2 h at room temperature protected from light. The coverslips were washed with 1x PBS, 3 times/10 min, mounted in VectaShield medium (Invitrogen) and analyzed under confocal microscopy (Leica TCS SP5 AOBS).

#### Real Time qPCR

To evaluate the expression levels of VEGF-A, U87MG cells were cultured and mRNA was extracted with TRIzol (Life Technologies) and precipitated in ethanol. Complementary DNA (cDNA) was synthesized from 1 μg of total RNA, using the Multiscribe kit (Applied Biosystem) according to the manufacturer's instructions. Quantitative PCR was performed in triplicate, using the Power SYBR Green Master Mix reagent (AppliedBiosystem) and VEGF-A forward primer: GAGTCCAACATCACCATGCA reverse primer: ACGCTCCAGGACTTATACCG. Relative quantification was performed using the ΔΔCt method, normalized to the expression of the GAPDH gene, forward primer: CCCATCACCATCTTCCAGGA; reverse primer: ATGATGACCCTTTTGGCTCC.

#### Scratch Assay

HBMEC cells, obtained from the Rio de Janeiro Cell Bank (BCRJ), were cultured in DMEM F12 medium (Dulbecco's Modified Eagle Medium/Nutrient Mixture F-12) supplemented with 10% Fetal Bovine Serum (FBS) and 0.1 mg/ml penicillin/streptomycin (PS) in 25 cm^2^ flasks at 37 °C in a 5% CO2 incubator until reaching 80 to 90% of confluence. The cells underwent trypsinization, were centrifuged at 1600 rpm for 7 min, resuspended in 1x PBS, counted, plated at the density of 2.5 × 10^4^ cells per well in a 24-well culture plate in DMEM F12 medium supplemented with 10% FBS and 0.1 mg/ml PS. After 24 h at 37 °C, 5% CO2, the FBS-enriched medium was removed, the cells were washed 3 times with 1x PBS and DMEM F12 medium enriched only with 0.1 mg/ml PS (DMEM F12/PS) was added. The cells were further conditioned for 24 h in the incubator under the same temperature and CO2 conditions described above. Using a P1000 pipette tip, a straight scratch was made in the cell monolayer in the central region of each well, taking care to produce scratches of similar sizes between the wells. Subsequently, the medium was removed, the cells were washed 3 times with 1x PBS and 500 μl of DMEM F12/PS medium was added to the 4 wells designated as controls. In the other wells, 500 μl of DMEM F12/PS medium enriched with the secretome of GBM cells treated with DMSO (4 different DMSO treated secretomes) or iHDAC (4 different iHDAC-treated secretomes) were added. Photographs were taken every 2 h using optical microscopy. Between the time intervals, the plate was conditioned at a controlled temperature of 37 °C and 5% CO2.

#### Tubulogenesis Assay

HBMEC cell line was cultured at the same conditions as above described. The culture plate and P200 pipette tips were stored in the freezer the night before the assay to prevent the gel from solidifying during the process and the Matrigel was kept on ice in a cold room to thaw without solidifying. In a 48-well culture plate, 120 μl of Matrigel was added to each well and kept in a CO2 incubator at 37 °C and 5% CO2 for 30 min. Then, 3 × 10^4^ cells were plated and DMEM F12/FBS+PS was added to the control group. The secretome obtained from the same groups used in the scratch assay were added and photographs were taken every 4 h using optical microscopy. During the intervals, the plate was kept in a CO2 incubator at a controlled temperature of 37 °C and 5% CO2.

#### Image Analysis

Images were acquired in a confocal microscope at a 40× magnification for 2D and 3D cell culture. For 2D cell culture the fluorescence intensity was measured by calculating the mean of the fluorescence divided by the mean of the number of nuclei in each image. 3D quantitative analysis was performed using Total Cell Fluorescence (TCF) of the spheroid, according to the following calculation: TCF = spheroid CTCF/spheroid area ∗ number of stacks.

#### Experimental Design and Statistical Rationale

The effect of iHDAC treatment on the U87MG GBM secretome analysis was conducted after 72 h of treatment with 100 nM Trichostatin A or DMSO as control. The iHDAC and control secretomes were obtained for two biological replicates of each condition and triplicate LC-MS/MS runs were conducted for each sample. To check the reproducibility of the replicates we use Pearson correlation between the three technical runs of each sample (Supplemental Fig. S1). Then we filtered the proteins identified between the two biological replicates of TSA1 and TSA2 and the two biological replicates of DMSO1 and DMSO2, that were used to compare the differentially secreted proteins (Supplemental Fig. S2). Statistical analysis was carried out in Perseus v 1.6.10.50 and R. Protein area values were converted to log2 scale and normalized by subtracting the median of the sample distribution. Proteins with<50% valid values in each group (DMSO and TSA) were removed from the analysis. The remaining proteins were subjected to missing value imputation using the default parameters (width 0.3, down shift 1.8) of the Perseus Imputation tool. GraphPad Prism (v6.0, La Jolla, CA) was used for ordinary One-Way or Two-Way ANOVA analysis. If the ANOVA produced a significant result, post hoc pairwise comparisons were tested for significance in which the value was adjusted (Padj< 0,05) by Tukey’s method for multiple comparisons inside each group. Results are presented as mean ± SEM and statistical relevance was defined as *p* < 0,05.

## Results

### HDAC Activity is Necessary for GBM Secretome Molecular Signature and Targets the Extracellular Matrix–Related Proteins

**Fig. 1:**
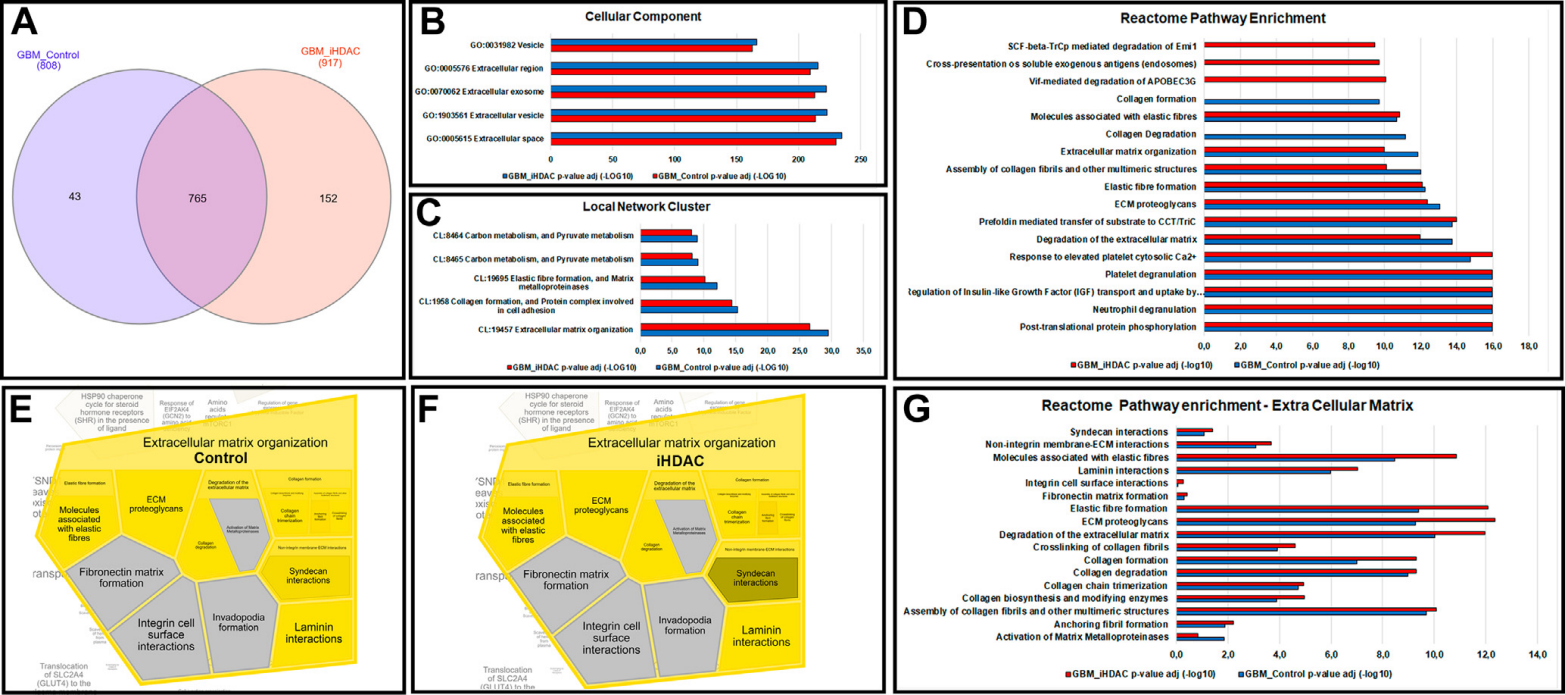
**HDAC activity is necessary for GBM secretome molecular signature and targets the extracellular matrix–related proteins.***A*, venn diagram showing the number of proteins found only in control group (*orange*), in the iHDAC group (*blue*), and common proteins (intersecting regions). The top five components of cellular component (*B*) and (*C*) local network cluster from STRING represented by the proteins found in the secretomes were selected and ranked according to the FDR *p* value showing the most enriched components in the secretome of both control and iHDAC. *D*, REACTOME pathway enrichment analysis of the 808 proteins from the control (*blue* bars) and the 917 proteins from iHDAC (*red* bars); 17 statistically significant pathways (*p* < 0.05) are listed and the *p* values for pathway enrichment are adjusted to -log10 (padj). *E*, Reacfoam or Voronoi graph depicts only the components of the control and (*F*) iHDAC secretomes that belong to the extracellular matrix organization according to REACTOME classification. Each polygon represents extracellular matrix organization sub pathways and the area of each polygon is directly related to the number of proteins identified in the secretome that map in each sub pathway. *G*, enrichment analysis of extracellular matrix organization sub pathways found in Reacfoam that are significantly enriched (*p* < 0.05; *p* value adjusted to -log10 (padj)). *Blue* bars indicate extracellular matrix organization sub pathways enrichment in control and *red* bars indicate the enriched in the iHDAC secretome.

Although the effects of iHDACs in cancer cells (27) and the secretome of GBM have been extensively characterized in the literature (28, 29) the putative effects of iHDACs on the repertoire of secreted mediators still remain unexplored. Due to the relevance of the epigenome as a druggable target for GBM we proposed to characterize, for the first time in the literature, the effects of iHDACs on the secretome of GBM focusing on the ECM associated proteins, the tumor Matrisome. For this we have used U87MG GBM cells for pharmacologically knockdown HDAC activity *in vitro*. U87MG cells were cultivated for 72 h in the presence of DMSO (control group) or iHDAC (Trichostatin A 100 nM) and the total conditioned medium was concentrated in AMICON tubes giving rise to the secretome. Cell death and cell cycle analysis using flow cytometry were performed to monitor cellular viability along the 72 h of experiment and did not show significant difference between control and iHDAC treated cells (Supplemental Fig. S3). NANOLC MS/MS analysis identified a total of 1145 proteins in the secretome of control and iHDAC groups (Supplemental Table S1). While 43 proteins were only identified in the control group, 152 proteins were only identified in the iHDAC group. Besides that, 765 proteins were common to both groups (Fig. 1*A*; Supplemental Table S2). Aiming to compare the repertoire of GBM secreted molecules with the health cell counterpart, the secretome of normal human astrocyte was used to better understand the main proteomic similarities and differences between normal and transformed astrocytic cells (30). While only 1 protein was common between GBM control and normal human astrocytes secretomes, 13 proteins were common between GBM iHDAC and astrocytes secretomes (Supplemental Fig. S4*A*; Supplemental Table S3). In addition, 143 proteins were common between GBM control, GBM iHDAC and astrocytes. Among them 5 proteins were downregulated and 7 proteins were upregulated in iHDAC (Supplemental Fig. S4*B*; Supplemental Table S3). The set of proteins in control and iHDAC secretome was subjected to protein-protein interaction (PPI) network analysis using STRING with confidence values >0.7 (high confidence). The network interaction protein map in the control group showed 901 nodes and 4754 edges while the iHDAC group showed 805 nodes and 3967 edges with a PPI enrichment *p* value < 10^16^ for both groups. The set of 322 proteins from normal human astrocytes presented a network interaction protein map with 322 nodes and 990 edges and a PPI enrichment *p* value < 10^16^. The top 5 components of cellular component and local network cluster from STRING were selected and ranked according to the FDR *p* value (Fig. 1, *B* and *C*; Supplemental Fig. S5, *A* and *B*). This analysis confirmed that proteins belonging to the extracellular space-related cellular components and extracellular matrix organization were the most enriched components in the secretome of both control and iHDAC as well as in the secretome of normal human astrocytes. UNIPROT analysis corroborated this finding and showed that 381 proteins (33,2%) displayed signal peptide, which is a hallmark of secreted proteins (Supplemental Table S4).

To better understand how the proteins identified in the experimental groups were distributed in signaling pathways we performed a pathway enrichment analysis using Reactome (Fig. 1*D*; Supplemental Table S5). Protein profiling resulted in shortlisting of 17 enriched pathways (*p* < 0,05) observed in control and iHDAC treated secretome. Among them 12 signaling pathways were common to both groups (neutrophil degranulation, regulation of insulin-like growth factor (IGF), transport and uptake by insulin-like growth factor binding proteins (IGFBPs), platelet degranulation, response to elevated platelet cytosolic Ca2+, degradation of the extracellular matrix, prefoldin mediated transfer of substrate to CCT/TriC, ECM proteoglycans, elastic fiber formation, assembly of collagen fibrils and other multimeric structures, extracellular matrix organization and molecules associated with elastic fibers), 3 were present only in control group (post-translational protein phosphorylation, collagen degradation and collagen formation) and 3 were present only in iHDAC group (Vif-mediated degradation of APOBEC3G, cross-presentation of soluble exogenous antigens (endosomes) and SCF-beta-TrCp mediated degradation of Emi1). Interestingly, normal human astrocyte Reactome Pathway Enrichment analysis showed that 9 pathways were common to control and iHDAC (Post-translational protein phosphorylation, Regulation of Insulin-like Growth Factor (IGF) transport and uptake by Insulin-like Growth Factor Binding Proteins (IGFBPs), Platelet degranulation, Response to elevated platelet cytosolic Ca2+, Degradation of the extracellular matrix, ECM proteoglycans, Elastic fiber formation, Assembly of collagen fibrils and other multimeric structures, Extracellular matrix organization). Out of these 9 Reactome Pathways analyzed in normal astrocytes, 8 presented a decreased enrichment when compared to GBM control and iHDAC treated (Supplemental Fig. S5*C*). Next, we conducted Reactome enrichment analysis to clarify the most relevant biological process targeted by iHDAC treatment. It was interesting to note that 47% (8/17) of all common signaling pathways was related to ECM organization with the pathway enrichment analysis showing that the higher enrichment in iHDAC group was for ECM proteoglycans (*p* < 4,3 × 10^−3^) while the higher enrichment in control group was for degradation of the extracellular matrix (*p* < 9,8 × 10^−11^) (Fig. 1, *E*–*G*). Although in normal human astrocytes the higher enrichment was observed in extracellular matrix as in the control the group, it was comparatively less enriched (*p* < 10^−7^) (Supplemental Fig. S5, *D* and *E*).

Thus, we conclude that the secretome of GBM cells treated with iHDAC displayed both qualitative and quantitative differences if compared to the control and to the normal human astrocyte secretomes. The *in silico* enrichment analysis using STRING and Reactome confirmed that the most enriched biological process and cellular components are related to protein secretion and to the extracellular space. Local network cluster and pathway enrichment analysis showed that ECM-related proteins are the specific target for iHDAC treatment.

### HDAC Activity Inhibition Modifies the Abundance of Core Matrisome, Matrisome-Associated, ECM-Affiliated Proteins, and Proteoglycans in the Glioblastoma Secretome

**Fig. 2:**
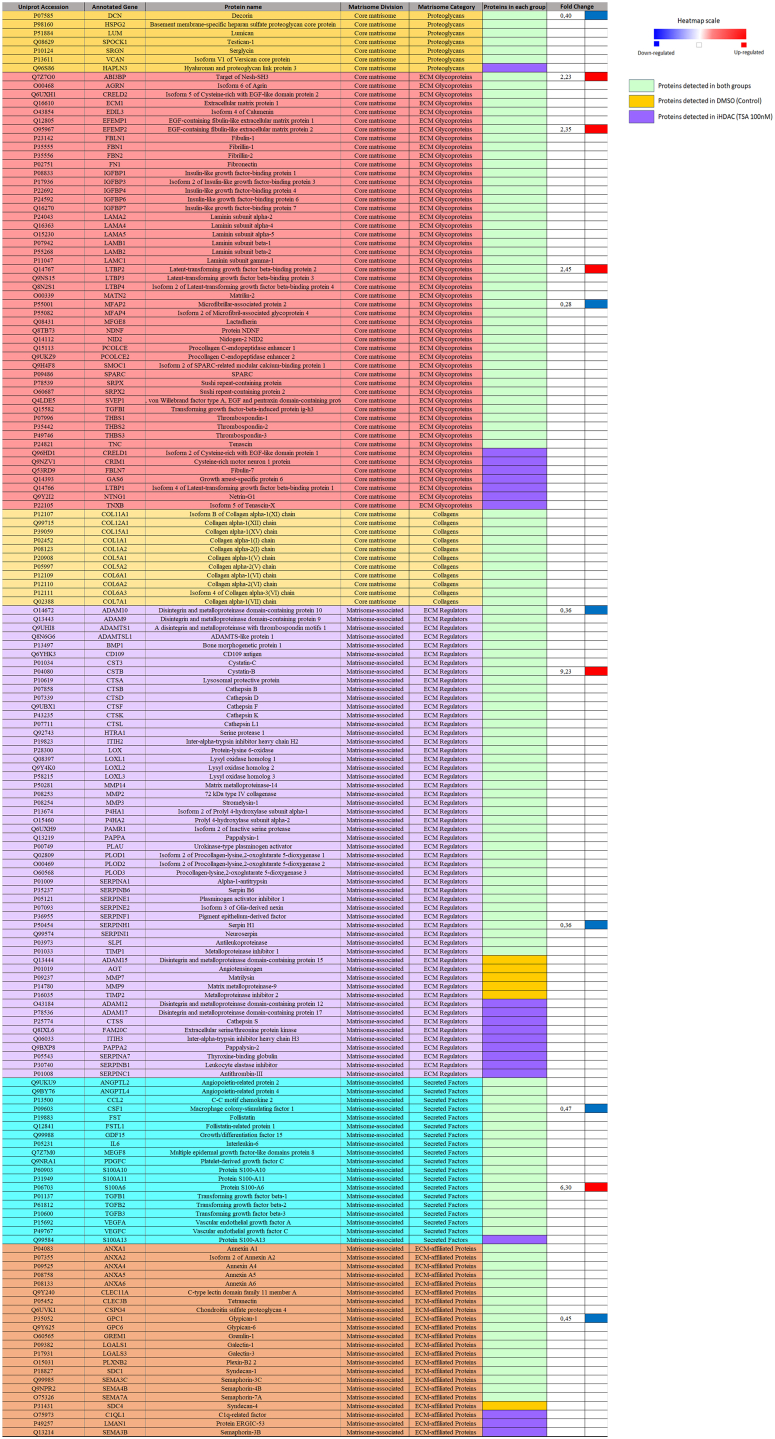
**HDAC activity inhibition modifies the abundance of core matrisome, matrisome-associated, ECM-affiliated proteins, and proteoglycans in the U87MG GBM secretome.***A*, the matrisome classification system was used to classify the 808 proteins in the control and the 917 proteins in the iHDAC secretomes and showed that 124 proteins were grouped in core-matrisome (60/142 = 42,25%), collagen division (11/142 = 7,74%), ECM-glycoprotein (43/142 = 29,57%), proteoglycan (6/142 = 4,22%), matrisome-associated (82/142 = 57,74%), ECM-affiliated proteins (19/142 = 13,38%), secreted factors (18/142 = 12,67%), ECM regulators (45/142 = 31%) in control secretome. In the iHDAC secretome, 157 proteins fit in the matrisome classification system and were grouped in core-matrisome (68/157 = 43;31%), collagen division (11/157 = 7%), ECM-glycoprotein (50/157 = 35%), proteoglycan (7/157 = 4,45%), matrisome-associated (89/157 = 56,68%), ECM-affiliated proteins (21/157 = 13,37%), secreted factors (19/157 = 12,10%), ECM regulators (49/157 = 31%). The heatmap and fold change indicate the proteins that were upregulated (*red*) or downregulated (*blue*) in iHDAC secretome. *Yellow* indicates the protein that were identified only in the control secretome, *purple* indicate the proteins identified only in iHDAC secretome, and *green* indicates the proteins that are common to both secretomes but did not have the abundance significantly altered upon iHDAC treatment.

The ECM in the brain is largely composed by fibrous proteins, glycosaminoglycans, proteoglycans and glycoproteins comprising ∼20% of the total mass in the brain (31, 32, 33, 34, 35). During gliomagenesis the ECM exerts an active role in tumor cell growth, proliferation, invasion, migration and angiogenesis being extensively remodeled by degradative enzymes secreted by the tumor cells (36). Due to the key role that ECM plays during invasion related GBM cell signaling, a better understanding on the effects that iHDAC have on the ECM deposited by GBM cells may improve the search for new druggable targets for GBM therapies and patient management. In this sense we performed the analysis of the secreted proteins using the Matrisome Classification System aiming to categorize the whole set of proteins present in the secretome of control and iHDAC groups. We observed that a total of 142 out of 808 (17,5%) of the proteins identified in the control secretome, and 157 out of 917 (17,1%) of the proteins identified in the iHDAC secretome, were ECM-associated proteins, involved in the organization and composition of the ECM (Fig. 2). In the normal human astrocytes secretome we observed 79 out of 322 ECM-associated proteins (24,5%) (Supplemental Table S6). More specifically, 142 proteins in the control group fit in the Matrisome Classification System (Core-Matrisome 60/142 = 42,25%; Collagen division 11/142 = 7,74%; ECM-Glycoprotein 43/142 = 29,57%; Proteoglycan 6/142 = 4,22%; Matrisome-associated 82/142 = 57,74%; ECM-affiliated Proteins 19/142 = 13,38%; Secreted Factors 18/142 = 12,67%; ECM Regulators 45/142 = 31%) (Fig. 2).

In the iHDAC 157 proteins fit in the Matrisome Classification System (Core-matrisome 68/157 = 43;31%; collagen division 11/157 = 7%; ECM-Glycoprotein 50/157 = 35%; Proteoglycan 7/157 = 4,45%; Matrisome-associated 89/157 = 56,68%; ECM-affiliated Proteins 21/157 = 13,37%; Secreted Factors 19/157 = 12,10%; ECM Regulators 49/157 = 31%) (Fig. 2).

In the normal human astrocyte 77 proteins fit in the Matrisome Classification System (Core-matrisome 45/77 = 58,4%; collagen division 9/77 = 11,6%; ECM-Glycoprotein 33/77 = 42,8%; Proteoglycan 3/77 = 3,8%; Matrisome-associated 32/77 = 41,5%; ECM-affiliated Proteins 5/77 = 6,4%; Secreted Factors 12/77 = 15,5%; ECM Regulators 15/77 = 19,4%) (Supplemental Table S6). It was possible to note that normal human astrocytes presented an enrichment in core-matrisome associated proteins and more specifically in ECM-glycoproteins if compared to the GBM Matrisome (Supplemental Fig. S6).

To better characterize the differences in the abundance of Matrisome classified protein between control and iHDAC groups, quantitative analysis was performed and presented using a heat map (Fig. 2; Supplemental Table S7). We observed that 5 proteins were significantly up regulated (red bars) and 6 proteins were significantly down regulated (blue bars) in the set of the Matrisome classified proteins in iHDAC secretome. Among the up regulated proteins, we observed 3 Core Matrisome (ECM-Glycoprotein Target of Nesh-SH3, Q7Z7G0, *p* value = 0.02 and FC = 2,2; EGF-containing fibulin-like extracellular matrix protein 2, O95967, *p* value = 0.042 and FC = 2,3; Latent-transforming growth factor beta-binding protein 2 Q14767, *p* value = 0.045 and FC = 2,4), 2 Matrisome-Associated (ECM-regulator Cystatin B, P04080, *p* value = 0.0018 and FC = 9,2); Secreted Factor S100-A6, P06703, *p* value = 0.00052 and FC = 6,3). Among the down regulated proteins, we observed 1 Core Matrisome (Proteoglycan Decorin, P07585, *p* value = 0.036 and FC = 0,04), 1 Core Matrisome (ECM-Glycoprotein Microfibrillar-associated protein 2 MFAP2, P55001, *p* value = 0.0014 and FC = 0,28), 4 Matrisome-associated (ECM Regulators: Disintegrin and metalloproteinase domain-containing protein 10 ADAM10, O14672, *p* value = 0.0011 and FC = 0,35; SERPINH1, P50454, *p* value = 0.00031 and FC = 0,3; Secreted Factors: Macrophage colony-stimulating factor 1 CSF1, P09603, *p* value = 0.028 and FC = 0,4.08; ECM-affiliated Protein Glypican1, P35052, *p* value = 0.0051 and FC = 0,4). We also highlight 5 proteins that were detected only in control and 21 proteins that were detected only in iHDAC treated secretome. In control secretome we detected 4 Matrisome-associated ECM Regulators (Disintegrin and metalloproteinase domain-containing protein 15, ADAM15, Q13444; Angiotensinogen, AGT, P01019; Matrilysin, MMP7, P09237; Matrix metalloproteinase-9, MMP9, P14780; Metalloproteinase inhibitor 2, TIMP2, P16035) and 1 Matrisome-associated (ECM-affiliated Protein Syndecan-4, SDC4, P31431). In contrast, in the iHDAC secretome we detected 8 Core Matrisome (1 Proteoglycan: Hyaluronan and proteoglycan link protein 3, HAPLN3, Q96S86; 6 ECM Glycoproteins: Isoform 2 of Cysteine-rich with EGF-like domain protein 1, CRELD1, Q96HD1; Cysteine-rich motor neuron 1 protein, CRIM1, Q9NZV1; Fibulin-7, FBLN7, Q53RD9; Growth arrest-specific protein 6, GAS6, Q14393; Isoform 4 of Latent-transforming growth factor beta-binding protein 1, LTBP1, Q14766; Netrin-G1, NTNG1, Q9Y2I2; Isoform 5 of Tenascin-X, TNXB, P22105), 13 Matrisome-associated (ECM Regulators: Disintegrin and metalloproteinase domain-containing protein 12, ADAM12, O43184; Disintegrin and metalloproteinase domain-containing protein 17, ADAM17, P78536; Cathepsin S, CTSS, P25774; Extracellular serine/threonine protein kinase, FAM20C, Q8IXL6; Inter-alpha-trypsin inhibitor heavy chain H3, ITIH3, Q06033; Pappalysin-2, PAPPA2, Q9BXP8; Thyroxine-binding globulin, SERPINA7, P05543; Leukocyte elastase inhibitor, SERPINB, P30740; Antithrombin-III, SERPINC1, P01008; Secreted Factor: Protein S100-A13, S100A13, Q99584; ECM-affiliated proteins: C1q-related factor, C1QL1, O75973; Protein ERGIC-53, LMAN1, P49257, Semaphorin-3B, SEMA3B, Q13214). Among the 77 proteins in the normal human astrocyte secretome that fit in the Matrisome classification system, we observed that 48 proteins were common to the GBM Matrisome (Supplemental Table S6). Among them we detected 4 proteins whose abundance was significantly down regulated in the iHDAC secretome (MFAP-2, CSF-1, GPC1, DCN), one protein whose abundance was up regulated (LTBL2) and 4 proteins detected only in the iHDAC secretome (Supplemental Fig. S6*C*).

Thus, we conclude that HDAC activity is necessary to modulate the abundance of core matrisome, matrisome-associated, ECM-affiliated proteins and proteoglycans in the GBM U87MG secretome and that normal human astrocytes Matrisome displays a different constitution if compared to transformed astrocytes.

### Differentially Regulated GBM Matrisome Proteins Harbor Missense Mutations and are Associated to Glioma Pathophysiology and Clinical Features

To better understand the relevance of the Matrisome associated proteins characterized in this work for GBM pathophysiology, we used the cBioPortal and the catalog of somatic mutations in cancer (COSMIC) to integrate clinical data with the Matrisome proteins identified. For this, we chose a study previously published that analyzed a dataset comprising an array-based molecular profiling of 1122 patients newly diagnosed with low grade or high-grade glioma (37). To search in the database, we used the proteins found in the secretome whose genes were mutated in Ceccarelli and collaborators (2016) (ABI3BP, EFEMP2, LTBP2, ADAM10, DCN, SERPINH1, ADAM15, MMP7, MMP9, CRELD1, CTSS, GAS6, HAPLN3, ITIH3, LTBP1, PAPPA2, SERPINA7, TNXB). We found 43 patients out of 1122 harboring mutations in these genes: 65,1% were male and 30,2% were female and the Glioblastoma Neoplasma Histologic Type (46,5%), histological grade 4 (G4, 46,5%), Pro Neural transcriptome subtype (32,6%), IDH wild type (58,1%), with methylated MGMT promoter (53,5%) and short overall survival (<15 months) were the features more frequent associated with patients harboring mutations in differentially regulated Matrisome associated proteins (Supplemental Table S8). Mutations and gene amplification were the genomic alterations observed in genes coding Matrisome proteins in these patients. Interestingly, missense mutations in this set of Matrisome proteins was the most frequent type of mutation observed among patients diagnosed with different types of tumors, including carcinoma, primitive neuroectodermal tumor-medulloblastoma, glioma, lymphoid neoplasm, hematopoietic neoplasm and malignant melanoma (Supplemental Fig. S8, *A*–*C*).

**Fig. 3:**
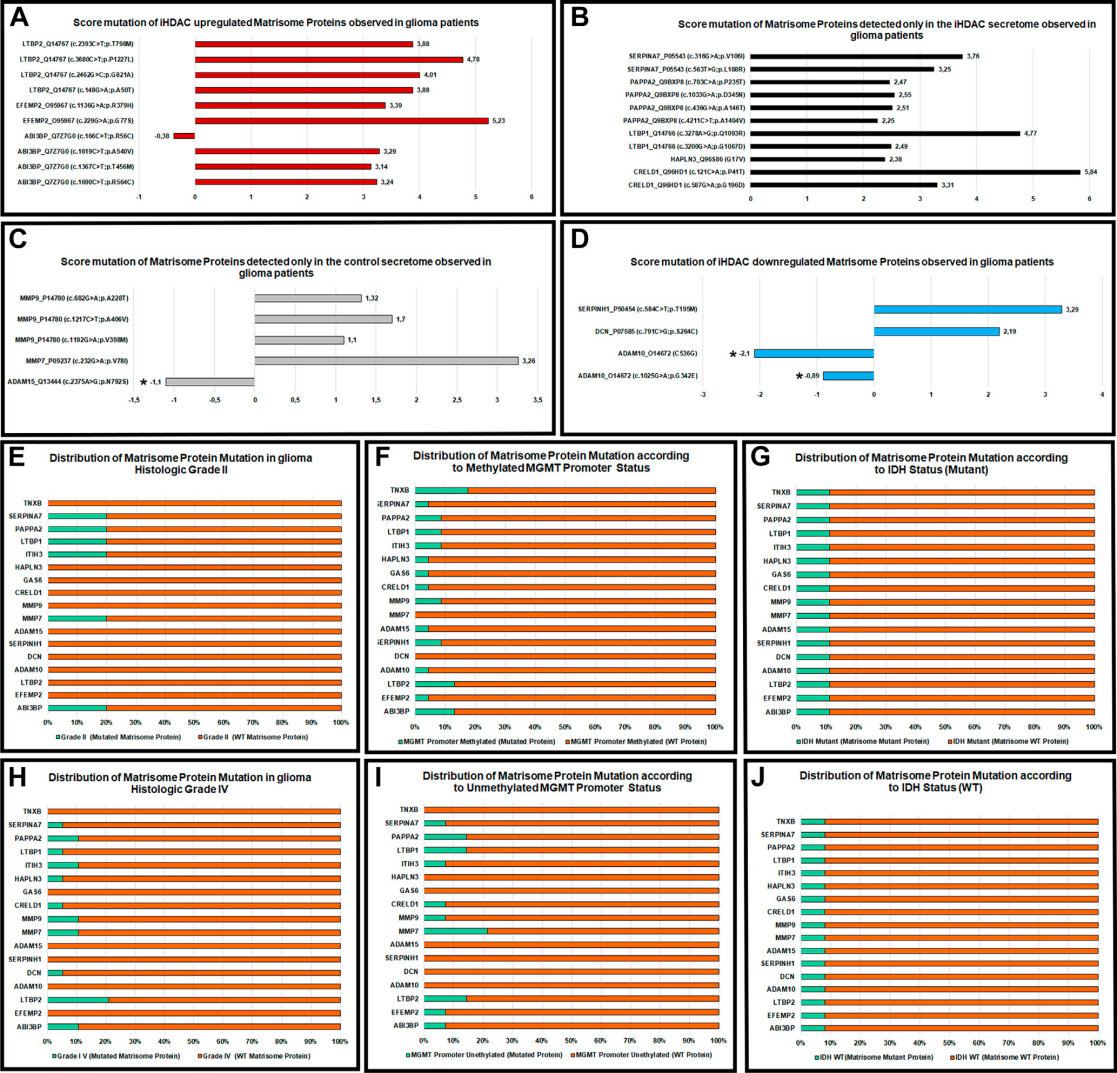
**The proteins characterized in the secretome of GBM harbor mutations and are related to glioma pathophysiology and clinical features.** Markov models (FATHMM) through the Analyze Cancer-Associated Variants tool using a prediction threshold = −0.75 was employed to analyze functional effects of matrisome proteins missense mutations in iHDAC upregulated matrisome proteins (*A*), proteins detected only in the iHDAC secretome (*B*), proteins detected only in the control secretome (*C*), or up down regulated in the iHDAC secretome (*D*). cBioPortal was used to evaluate 43 patients out 1122 newly diagnosed with low grade or high grade glioma. The cBioPortal dataset comprised an array-based molecular profiling and only the patients harboring mutations for genes coding for matrisome proteins were analyzed. Matrisome proteins harboring mutations were correlated with low (*E*; grade 2) or high (*H*) grade gliomas, methylated (*F*) or unmethylated (*I*) MGMT promoter status, mutant (*G*) or WT (*J*) IHD status.

Aiming to provide a better understanding on the functional effects of Matrisome proteins missense mutations and their putative clinical implications for glioma pathophysiology, and separate disease-associated variants from common polymorphism as well as deleterious variants from neutral variants, we employed functional analysis through hidden Markov models (FATHMM) through the Analyze Cancer-Associated Variants tool using a prediction threshold = −0,75. ADAM 10, a Matrisome protein downregulated in iHDAC, and ADAM 15, a Matrisome protein detected only in the control secretome, presented significant score for pathogenic mutation (Fig. 3, *A*–*D*), suggesting that these mutations might function as biomarkers for glioma grading.

While mutations in EFEMP2, ADAM10, SERPINH1, ADAM15, GAS6 and TNXB were detected only in patients with glioma grade 3, mutations in LTBP2, DCN, CRELD1 and HAPLN3 were observed only in patients with glioma grade 4, GBM (Fig. 3, *E* and *H*; Supplemental Table S8). Mutations in ADAM10, ADAM15, GAS6 and HAPLN3 were observed in patients harboring the methylated MGMT promoter status while mutation in CRELD1 was observed associated only to the unmethylated MGMT promoter status (Fig. 3, *F* and *I*). Mutations in ADAM10, ADAM15 and GAS6 were associated to the mutant IDH status while mutations in DCN and CRELD1 were associated to the wild type IDH status (Fig. 3, *G* and *J*; Supplemental Table S8). Remarkably, both ADAM10 G342E (score = −0,81) and ADAM15 N792S (score −1,1) missense pathogenic mutation were associated to low grade glioma, methylated MGMT promoter status and mutant IDH status, which correspond to a positive prognosis in glioma patients. This finding suggests that these 2 Matrisome proteins differentially regulated between control and iHDAC secretomes may emerge as promising biomarkers for glioma progression follow up.

### The Secretome of iHDAC-Treated U87MG GBM Cells Presents the Angiomatrix Signature Disrupted and Impairs Endothelial Cell Behavior

**Fig. 4:**
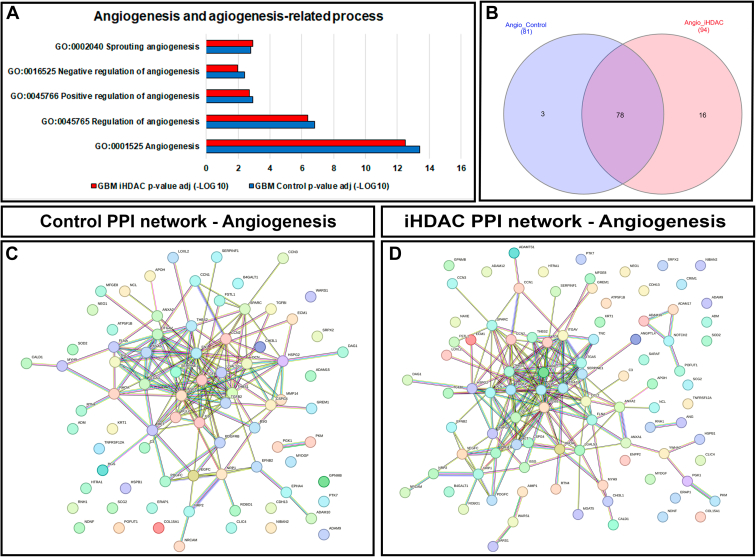
**HDAC activity inhibition modifies the abundance of angiogenesis and angiogenesis-related biological process.***A*, The top five components of angiogenesis and angiogenesis-related biological process were selected and ranked according to the FDR *p* value, which indicated that angiogenesis (GO0001525), regulation of angiogenesis (GO0045765), positive (G0045766) and negative regulation of angiogenesis (GO0016525), and sprouting angiogenesis (GO0002040) were consistently enriched in the secretome of control (*blue* bar) and iHDAC group (*red* bar). *B*, venn diagram illustrating that proteins belonging to angiogenesis and angiogenesis-related biological process were observed in control (81 proteins) and iHDAC (94 proteins) secretomes. *C*, STRING PPI network representation of (GO0001525) angiogenesis-related proteins in control and (*D*) iHDAC secretomes.

It is well known that the Matrisome associated proteins identified so far may play critical roles on GBM biology and have been described as active players during tumor cell growth, proliferation, invasion, migration and angiogenesis (38). During GBM growth, the rapid cellular proliferation leads to nutrient deprivation which in turn culminates in the enhancement of ECM remodeling by MMPs and angiogenesis. In fact, anti-angiogenic GBM therapy, Bevacizumab that targets the vascular endothelial growth factor (VEGF)-A, has been employed for the treatment of GBM without significant enhancement in the overall survival. For this reason, new therapeutic approaches targeting GBM neoangiogenesis could increase antitumor immune response for example (39, 40, 41). Aiming to better understand whether iHDAC treatment could modulate the Core Matrisome, Matrisome-associated, ECM-affiliated proteins and Proteoglycans that underly the mechanisms involved in GBM angiogenesis, we went to characterize the GBM AngioMatrix (42). Our set of results strongly suggest that the ECM components that are related to mechanisms that control angiogenesis might be the main target for iHDAC treatment. Firstly, we used STRING to identify the proteins in the secretome that belong to angiogenesis (GO00012525) and angiogenesis-related biological process. Using GO: Biological Process classification we detected the proteins included in angiogenesis (GO0045765), positive (G0045766) and negative regulation of angiogenesis (GO0016525), sprouting angiogenesis (GO0002040) and cell migration involved in sprouting angiogenesis (GO0002042) that were consistently enriched in the secretome of control (blue bar) and iHDAC group (red bar) (Fig. 4*A*). While 81 proteins belonging to angiogenesis and angiogenesis-related biological process were observed in control group (10% of total protein), 94 proteins were observed in the iHDAC secretome (10% total protein) (Fig. 4*B*; Supplemental Table S9). We observed that 78 proteins belonging to angiogenesis and angiogenesis-related biological process were common to both experimental groups and among of them, 4 were downregulated (ADAM10; SOD2; DCN, CLIC4) and 1 was up regulated in iHDAC group (; RHOA) (Supplemental Table S9). The PPI network established by common proteins that belong to angiogenesis and angiogenesis-related process is illustrated in Figure 4, *C* and *D*. In contrast, normal human astrocytes presented 44 out of 322 proteins (13%) belonging to angiogenesis (GO0045765), positive (G0045766) and negative regulation of angiogenesis (GO0016525) and sprouting angiogenesis (GO0002040) process with a significant FDR *p*-value (Supplemental Fig. S9, *A* and *B*). Next, we built the PPI network with confidence values >0.7 (high confidence) and observed that normal human astrocytes share 25 proteins with the angiogenic signature with GBM (Supplemental Fig. S9*C*).

**Fig. 5:**
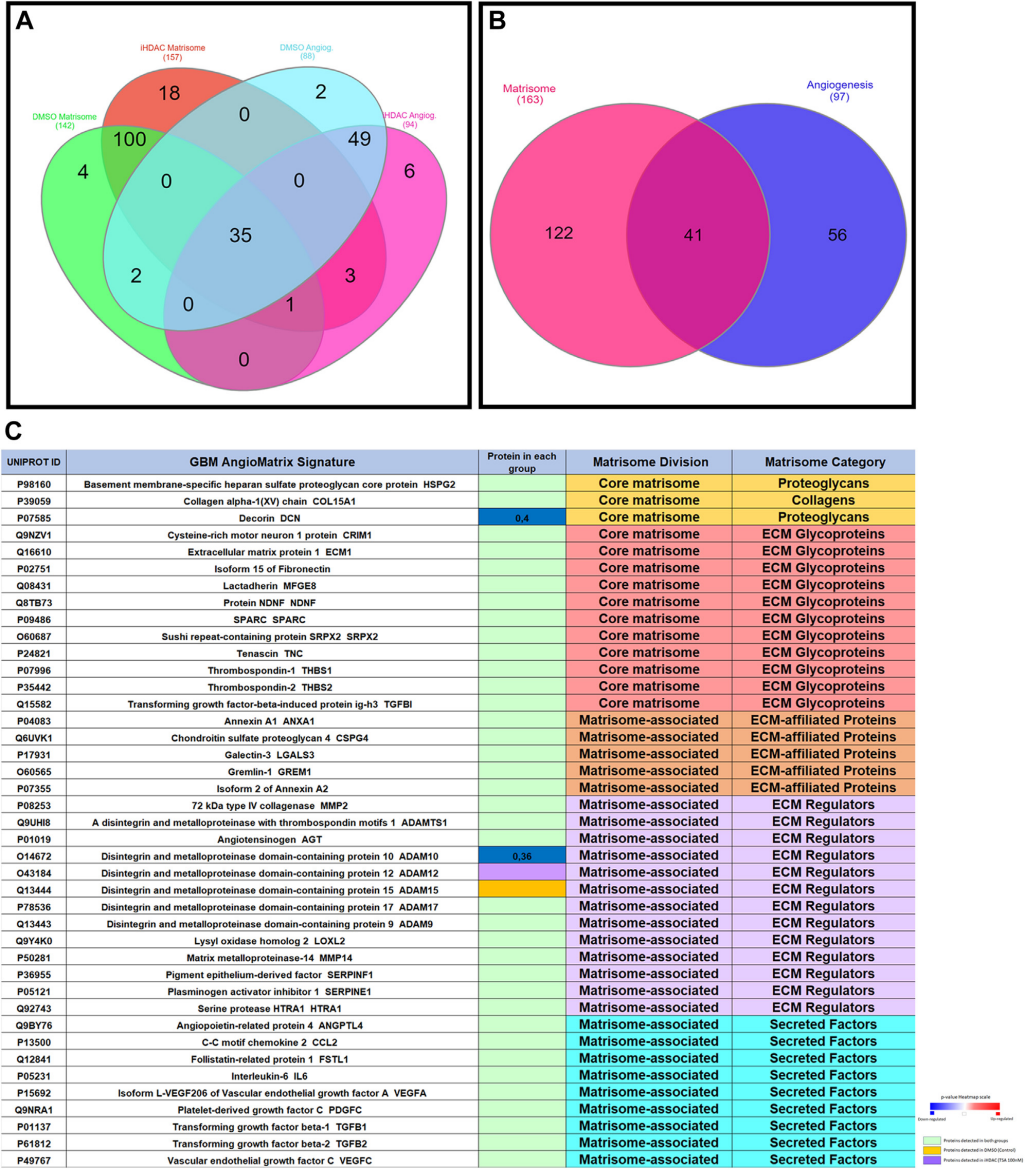
**The GBM Angiomatrix signature is disrupted upon iHDAC treatment.***A*, UNIPROT classification of proteins related to angiogenesis in control and iHDAC secretomes were merged with the matrisome classified proteins, and 33 proteins in control and 39 proteins in iHDAC secretomes were assigned to the AngioMatrix signature. *B*, the merge of the matrisome and angiogenic proteins from both experimental groups identified 122 proteins that belong to the matrisome, 56 proteins that belong to angiogenesis, and 41 proteins were shared between matrisome and angiogenesis. These 41 proteins were assigned as the AngioMatrix signature for both secretomes. *C*, table showing the proteins that belong to the AngioMatrix signature in the GBM secretomes. ADAM10 (matrisome associated; ECM regulator) and DCN (core matrisome; proteoglycan) were significantly downregulated in iHDAC (Fold Change; *blue box*), ADAM15 (matrisome associated; ECM regulator) was detected only in control (*yellow box*), and ADAM12 (matrisome associated; ECM regulator) was detected only in iHDAC secretomes (*purple box*).

To establish the AngioMatrix signature in our secretome we used UNIPROT to obtain the whole set of proteins related to angiogenesis and afterward merged them with the Matrisome classified proteins (Fig. 5*A* and Supplemental Table S9). While in the control group this strategy retrieved 33 AngioMatrix proteins (4% of total protein), in iHDAC group we detected 39 AngioMatrix proteins (4% of total protein) (Fig. 5*A*). After this, we merged Matrisome and Angiogenic proteins from both experimental groups and retrieved 122 proteins that belong to the Matrisome, 56 proteins that belong to angiogenesis and 41 proteins shared between Matrisome and Angiogenesis that gave rise to AngioMatrix signature in the GBM secretome (Fig. 5*B*). Among them ADAM10 (Matrisome Associated; ECM regulator) and DCN (Core Matrisome; Proteoglycan) were significantly downregulated in iHDAC, ADAM15 (Matrisome associated; ECM Regulator) was detected only in control and ADAM12 (Matrisome Associated; ECM regulator) was detected only in iHDAC secretome (Fig. 5*C*). Using STRING database, we observed that this set of proteins significantly (FDR 0,0057) controls the extracellular matrix organization (GO:0030198) in the context of the regulation of vasculature development (GO:1901342). Although the AngioMatrix signature of normal human astrocytes was composed by a slightly higher number of proteins (18 out of 322; 5% of total proteins), ECM-regulators and ECM-affiliated proteins were less represented (Supplemental Fig. S9*E*). In addition, only DCN protein presented significant difference in fold change when compared to iHDAC secretome (Supplemental Fig. S9*F*). Although the normal astrocyte secretome share 13 proteins with the iHDAC secretome, only 1 protein (CRM1) belongs to the normal astrocyte AngioMatrix signature, indicating that the AngioMatrix of normal human astrocytes is not predicted to be a target for iHDACs. These findings corroborate the hypothesis that the ECM, in the context of angiogenesis and angiogenesis-related biological process, is the main target for iHDAC in the GBM secretome.

**Fig. 6:**
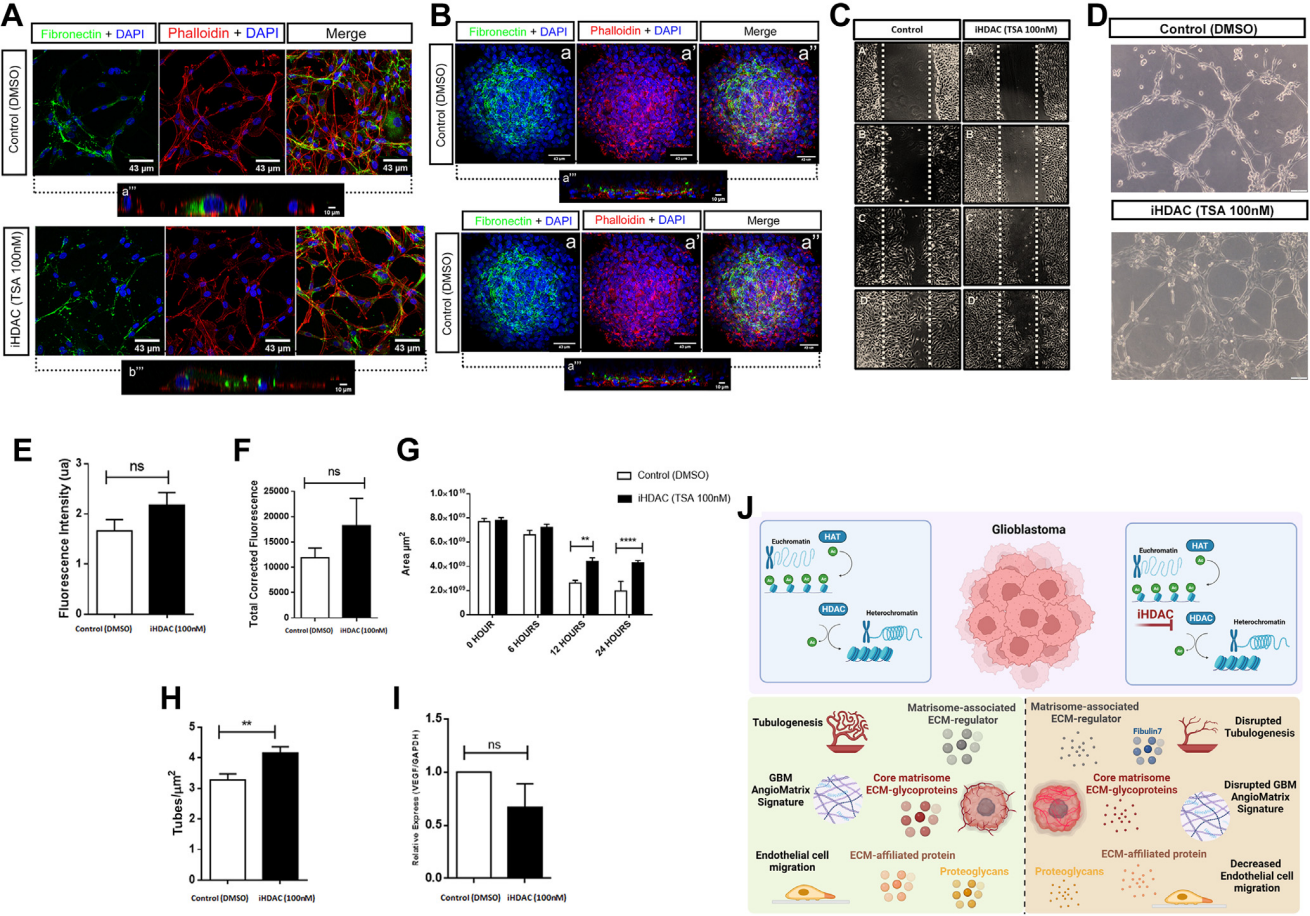
**iHDAC-treated U87MG secretome impairs endothelial cell behavior.***A*, confocal microscopy images showing the immunostaining pattern of fibronectin in 2D or (*B*) 3D U87MG cell cultures. *C*, illustrative images of scratch assay using control or iHDAC-treated secretomes on HBMEC endothelial cells at 0, 6, 12, 24 h upon secretomes incubation. *D*, tube formation assay was performed using control or iHDAC-treated secretomes on HBMEC endothelial cells. *E*, no significant difference was observed in fibronectin upon fluorescence quantification in 2D model. *F*, quantitative analysis of fibronectin performed using the total cell fluorescence (TCF) of the spheroid, where we used the following calculation: TCF = spheroid CTCF/spheroid area **×** number of stacks. *G*, quantification of the migratory capacity of endothelial cells. *H*, the number of tubes was quantified after 24 h of treatment. *I*, real time qPCR for VEGF was performed in U87MG cells from control or iHDAC treatment upon 72 h. *J*, working model: iHDAC activity inhibition modifies qualitatively and quantitatively the main components of the GBM angiogenic matrisome, the AngioMatrix, including matrisome-associated ECM regulators, core matrisome ECM glycoproteins, ECM-affiliated proteins and proteoglycans. These changes in the GBM AngioMatrix upon iHDAC treatment led to impaired endothelial cell migration and aberrant tubulogenesis in a fibronectin and VEGF-independent fashion. Quantitative analysis was performed through fluorescence intensity and normalized by the number of nuclei/images. Statistical analyzes were performed using two-Way ANOVA to compare differences between the cell lines from the same treatment group (*black* lines), where ∗*p* < 0.05; ∗∗*p* < 0.01; ∗∗∗*p* < 0.001; ∗∗∗∗*p* < 0.0001. Error bars correspond to SEM. Scale bar represents 43 μm.

To test whether the secretome of iHDAC treated GBM U87MG cells would impair endothelial cell behavior, scratch and tube formation assays were performed to investigate endothelial cell migration and tubulogenesis capacity upon exposure to secretomes obtained from control and iHDAC treated U87MG GBM cells. Because Fibronectin (FN) is a well-known regulator of endothelial cell migration on the ECM whose abundance was not significantly changed between control and iHDAC secretome, we decided to validate this finding aiming to rule out any putative FN effect on the scratch assay. In fact, using 2D and 3D U87MG cell culture assays we did not detect any significant difference in the FN immunostaining pattern between control and iHDAC cultures (Fig. 6, *A*, *B*, *E* and *F*). The scratch assay showed that endothelial cells treated with the iHDAC secretome displayed a significant decreased migratory capacity when compared to control secretome as the healing area was significantly increased after 24 h of assay (Fig. 6, *C* and *G*). Besides that, the *in vitro* tube formation capacity of endothelial cells was disrupted in the presence of iHDAC secretome (Fig. 6, *D* and *H*). Although VEGF-A had been detected in both secretomes without significant statistical differences, we performed a qPCR to rule out putative effects of VEGFA on the migratory and tubulogenesis capacity of endothelial cells observed in the assays (Fig. 6*I*). In fact, we did not detect significant differences in VEGF-A expression levels between control and iHDAC treated U87MG GBM cells.

This result corroborates the *in silico* analysis and supports the hypothesis that iHDAC mainly targets the components of the U87MG GBM cell secretome involved in angiogenesis and angiogenesis-related biological process, the AngioMatrix, in a FN and VEGF independent fashion.

## Discussion

In this work we provide evidence that the inhibition of HDAC activity disrupts the molecular signature of U87MG GBM cells secretome, focusing on the angiogenic Matrisome signature, the AngioMatrix, with critical functional impact on endothelial cell behavior and angiogenesis. iHDACs from different chemical classes including hydroxamates, cyclic peptides, short-chain aliphatic fatty acids and benzamides have been extensively tested against different malignant neoplasms (27, 43). Although the use of iHDACs for oncology has been approved only for T-cell lymphoma or for multiple myeloma, new iHDACs are being developed for clinical use, which highlights their potential for cancer treatment (44, 45, 46). To date the FDA-approved iHDACs for Peripheral T-cell lymphoma are Belinostat and Romidepsin, for Multiple Myeloma Panobinostat and for Cutaneous T-cell lymphoma Romidepsin and Vorinostat (SAHA) (43, 47). iHDACs have also been extensively studied using *in vitro* assays and different GBM cells. Among the main effects observed we highlight significantly reduced proliferation rates of GBM stem cells (48, 49) and GBM cells differentiation, increased cytotoxicity to anti-cancer drugs (48, 50) reduction of GBM cell invasion, migration, angiogenesis (51, 52) and autophagy (53).

However, there is a lack of knowledge on the participation of epigenetic mechanisms HDAC-dependent in controlling the abundance of ECM components in the context of GBM biology. Given that peritumoral infiltration is a hallmark of infiltrative gliomas such as GBM, the abundance of ECM components may emerge as an important biomarker for diagnosis and prognosis in GBM. In fact, several studies have already demonstrated the existence of consistent differences in ECM components between non-tumor brain and low- or high-grade gliomas (54).

Previous work from our group, using live cell imaging, demonstrated that two different iHDACs were implicate in a decrease in the U87MG cells migration and invasion rates, suggesting that iHDAC may control the secretion of mediators that regulate the migratory capacity of tumor cells through the interaction and degradation of components of the ECM contributing to GBM aggressiveness (15). Following the classification system established by (22), that proposed a classification system for the ECM constituents and their functions and gave rise to the concept of Matrisome, which includes the divisions Core Matrisome (Proteoglycans, ECM glycoproteins, collagens) and Matrisome associated components (ECM regulators, secreted factors, ECM affiliated proteins), we performed a critical analysis and classification of our *in silico* results to better understand the impact of iHDAC treatment on the GBM secretome. In addition to laminin, collagen and hyaluronic acid, among other components, GBM cells can secrete high amounts of different Core Matrisome and Matrisome associated components, such as MMP 2 and 9, a feature that has been linked to dynamic remodeling of the ECM and to a poor prognosis (55). Our *in silico* analysis showed that 4 ECM-regulators that are Matrisome-associated proteins Disintegrin and metalloproteinase domain-containing protein 10 (ADAM10, O14672), Matrilysin (MMP7, P09237), Matrix metalloproteinase-9 (MMP9, P14780) and Disintegrin and metalloproteinase domain-containing protein 15 (ADAM15, Q13444) were significantly down regulated in iHDAC treated secretome. Matrix metalloproteinases (MMPs) along with different members of adamalysins including ADAMs (A disintegrin and metalloproteases) and ADAMTS (A disintegrin-like and metalloprotease domain (reprolysin type) with thrombospondin type 1 motifs) have been largely implicated in GBM biology and are related to a poor prognosis (56). It has been shown that ADAM10 is overexpressed in high grade gliomas (57), its inhibition can suppress glioma cell proliferation, migration and invasion (58). In addition, ADAM10 selectively affects GBM stem cells (GSC) migration and promotes their migration and differentiation in a susceptible phenotype for drug treatment (59). MMP7 and MMP9 are related to ECM remodeling and transcripts of both MMPs are up-regulated in high grade gliomas (60).

Decorin (DCN, P07585), a Core Matrisome proteoglycan that was down regulated in iHDAC secretome, is another candidate as a biomarker for GBM aggressiveness. Although decorin, biglycan and serglycin were found up regulated in GBM samples, only Decorin levels were negatively associated with GBM patient overall survival, suggesting Decorin as a putative microenvironmental glycomarkers/targets for GBM diagnostics and therapy (61). Our *in silico* results are in line with these findings and argue in favor that the HDAC activity inhibition may downgrade tumor malignancy and may function as a druggable target for GBM clinical trials.

Another important finding in this work was the down regulation of the Matrisome Associated ECM-affiliated protein Glypican-1, a prognostic biomarker in glioma that belongs to the family of heparan sulfate proteoglycans (HSPGs) (62) that is overexpressed in glioma (63) and has been linked to a poor prognosis in GBM patients (64). It has been shown that the expression of Glypican-1 is increased by Annexin A2 through a positive feedback loop with c-Myc that ultimately promotes glioma cell proliferation (65). Although 5 different Annexins had been identified in both control and iHDAC secretomes (A1, P04083; A2, P07355-2; A4, P09525; A5, P08758; A6, P08133), none of them presented significant differences in the abundance between experimental groups. Given that Glypican-1 was downregulated in iHDAC secretomes, our findings agree with previous results published by our and other groups in which we showed that iHDAC treated U87MG cells displayed impaired proliferation [15].

Microfibrillar-associated protein 2 (MFAP2, P55001) was also down regulated in iHDAC secretome. It is a core matrisome protein that belongs to the ECM glycoproteins group which is involved in microfibrillar-assembly elastin production and signal transduction (66). MFAP2 is highly expressed in a wide range of malignant tumors including GBM and plays a key role in glioma cell migration, proliferation, angiogenesis and was related to malignant phenotypes and to a poor prognosis of patients with GBM (67). These results indicate that MFAP2 would emerge as a prognostic marker for gliomas and suggest its value as an epigenetic druggable target for GBM therapy.

Serpin (SERPINH1, P50454), another Matrisome associated protein and ECM-regulator, is a member of the superfamily of serine proteinase inhibitors with critical roles in anticoagulative/inflammatory system that has been validated in pre-clinical studies as a potent anti-tumor drug (68). In gliomas SERPINH1 overexpression was correlated to a poor overall survival and to a reduced progression free rate (69) due to the promotion of GBM invasion, angiogenesis and stemness (70). Conversely, SERPINH1 knockdown led to a significant decrease in GBM cell viability *in vitro* and *in vivo* as well as to a reduced microvessel density (71). In this sense, the down regulation of SERPINH1 in iHDAC secretome agrees with scratch assay in which we observed that iHDAC secretome led to a decrease in endothelial cell motility in a FN and VEGF independent fashion.

Another interesting finding of this work was the identification of Fibulin7 only in the iHDAC secretome as this secreted molecule displays antiangiogenic activity *in vivo* (72). It has been shown that Fibulin7 can inhibit neovascularization *in vivo* and that the C-terminal of Fibulin7 competes with VEGF by physically binding to VEGFR2. In addition, Fibulin7 competes with FN by the integrin α5β1 binding on the endothelial cell surface and reduces endothelial cell motility (72). Our *in silico* findings and *in vitro* assays are in line with these results previously reported in the literature and corroborate that the AngioMatrix is a relevant target of HDAC activity. In summary, given that 37 out of 163 Matrisome proteins (22%) were differentially regulated upon iHDAC treatment (6 proteins down regulated; 5 proteins up regulated; 21 proteins detected only in the iHDAC secretome and 5 proteins detected only in control secretome), we interpret that the Matrisome is a target for iHDACs and may benefit and/or improve current GBM clinical approaches.

Expanding the Matrisome concept, Langlois *et al.* (42), (2014) established the merge between the Matrisome and Angiogenesis related proteins and gave rise to the concept of AngioMatrix. In this model the authors have used the RIP1-Tag2 transgenic murine model to determine the set of Matrisome related genes that was up or down regulated upon the angiogenic switch to characterize the AngioMatrix signature in their model. In agreement Hawkins *et al.* (73) (2020) showed that Wnt/β-catenin signaling is involved in Ewing sarcoma progression by controlling the AngioMatrix signature in this tumor. Angiogenesis is a hallmark of cancer and is typical of high-grade gliomas such as GBM presenting aberrant morphological patterns with tortuous blood vessels that may impair drug delivery in the brain (74). In fact, GBM anti-angiogenic therapies have been put forward and approved for use by FDA. This is the case of Bevacizumab, a human monoclonal antibody that blocks vascular endothelial growth factor (VEGF-A) and ameliorates the main clinical symptoms without significant benefits on patient overall survival (75). It is well known that GBM secretes high amounts of VEGF, a feature that was characterized in control and iHDAC secretomes. Although the treatment with iHDAC did not significantly modify the abundance of VEGF-A and VEGF-C in the secretomes nor the enrichment of VEGF-associated signaling pathways in our *in silico* analysis, we observed that the iHDAC secretome disrupted endothelial cell behavior *in vitro*. Thus, we conclude that HDAC activity can modulate the AngioMatrix signature of the GBM secretome. Strikingly, ADMA10 and ADAM15 AngioMatrix proteins were the only ones that presented predictable missense pathogenic mutations, highlighting the AngioMatrix signature as a valuable source of biomarkers for GBM progression follow up. In this sense, druggable epigenetic therapies specifically designed to modulate the GBM AngioMatrix signature may emerge as promising targets for new approaches against this malignancy given that iHDAC treatment led to a significant decrease in the abundance of ADAM10 and to the absence of ADAM15 from GBM secretome.

We have expanded our analysis using normal human astrocytes secretome aiming to better characterize the main structural features of the AngioMatrix in normal human astrocytes (30) compared to their malignant counterpart. It was very interesting to note that the enrichment for cellular components related to cell secretion was lower in the secretome of normal human astrocytes if compared to control and iHDAC GBM secretomes. This difference indicates that tumorigenic cells present a higher secretion rate if compared to nonmalignant cells. In contrast, local cluster network and Reactome enrichment analysis showed that the extracellular matrix organization components were enriched in normal human astrocytes secretome if compared to control and iHDAC GBM secretomes, suggesting that normal astrocytes present a more organized ECM than malignant cells. In fact, it is very well known that tumor cells present an intense ECM remodeling corroborating our *in silico* analysis. The Matrisome components observed in the secretome of normal human astrocytes is quite different of the GBM one. It was interesting to note that core matrisome-associated proteins were enriched in normal human astrocytes, mostly due to the enrichment of ECM-glycoproteins. In contrast, the decrease of matrisome-associated proteins in the secretome of normal human astrocytes is mainly due to the decrease in the ECM regulators. It was very interesting to note that the normal human astrocyte Matrisome share 9 proteins that were differentially regulated upon iHDAC treatment or were detected only in the iHDAC secretome (DCN, MFAP2,CSF-1, GPC-1, LTBP2, CRM-1, GAS6, SERPINC1, FAM20C). Strikingly, 1 Matrisome protein detected only in the control secretome was observed in the normal human astrocyte secretome. We hypothesize that iHDAC treatment might control the abundance of Matrisome associated proteins related to downgrading of tumor cells. This interpretation is supported by previous work published by our group, in which we demonstrated, both *in vivo* and *in vivo* that iHDAC treated GBM cells confers low malignance characteristics to tumor cells (15). Our *in silico* strategy showed that normal astrocytes secrete a range of angiogenic proteins, which is in line with the literature (76). However, malignant astrocytes present a consistent enhanced capacity for secreting angiogenic proteins if compared to their normal counterparts (77). Although GBM and normal human astrocyte secretomes share 25 proteins related to angiogenesis, the enrichment for this process in the secretome of normal human astrocytes is lower than in GBM secretomes. In addition, the structure and composition of the AngioMatrix were found to be strikingly different between normal human astrocytes and GBM cells. Although normal human astrocytes and GBM present 5% of secretome proteins as components of the AngioMatrix, the set of proteins that characterize the AngioMatrix signature was quite different, indicating that the AngioMatrix might not be a target for iHDACs in normal human astrocytes.

## Conclusion

In conclusion, the present work demonstrates that Proteomic analysis of the GBM secretome is an important tool for studying the impact of druggable targets for brain tumor cells under specific conditions. iHDAC treatment significantly altered the molecular signature of the GBM secretome with critical impact on the angiogenic Matrisome, the AngioMatrix (Fig. 6*J*). The study of tumor secretome may emerge as a valuable strategy to better characterize tumor cells by their molecular signatures using a proteomic strategy. We believe that this approach can shed light on the identification of useful new biomarkers to be tested in the clinic in a non-invasive way, with great potential for translational medicine in GBM. In this sense, the study of the molecular signature of the GBM secretome may shed light on new strategies that may aid a proper diagnosis with positive impacts on clinical outcomes.

## Data Availability

All data generated or analyzed during this study are included in this published article as well its supplementary information files. Proteomic raw data can be found at ProteomeXchange identifier PXD022982.

## Supplemental data

This article contains supplemental data (22, 30).

## Conflict of interest

The authors declare that they have no conflicts of interest with the contents of this article.

## Supplementary Data

Supplemental Figures

Supplemental Table S1 Proteins identified by LC-MS / MS in the U87MG glioblastoma cell line. Table shows 1145 proteins identified by Orbitrap mass spectrometry. Columns A and B show the protein name (description) and the UNIPROT ID (accession). In columns C-L there is basic information about the identified proteins, such as molecular weight, number of peptides, transcriptional modifications, etc. the M-X columns shows the detected areas for each protein identified under the conditions: iHDAC (TSA) and Control. Samples were made with two biological replicates and three experimental runs were performed for each biological replicate.

Supplemental Table S2 Proteins analyzed by the Venn Diagram. The proteins were separated in different sheets. The first one shows the 765 proteins common to the two groups (DMSO and iHDAC), the second one shows the 43 proteins found only in the Control group and the third one shows the 152 proteins found only in the iHDAC Group, represented in Venn Diagram (Fig. 1*A*).

Supplemental Table S3 List of proteins analyzed from Kim *et al.* 2022.

Supplemental Table S4 Proteins analyzed for the presence of signal peptides. Proteins were subjected to a search in the UNIPROT database to track the presence of signal peptides. The first sheet shows proteins common to Control and iHDAC secretome and 323 proteins with signal peptides were observed. The second sheet shows 18 proteins that were observed only in the Control secretome. The third sheet shows 40 proteins that were observed only in the iHDAC group.

Supplemental Table S5 Proteins analyzed in Reactome Pathway. The first and second sheets show the UNIPROT access of the 808 Control proteins and the Reactome-enriched pathways, respectively. The third and fourth sheets show the UNIPROT access of the 917 proteins from the iHDAC group and the Reactome-enriched signaling pathways. The fifth and sixth sheets show the Extracellular Matrix Organization signaling pathways from REACTOME in Control and iHDAC secretomes, respectively. The seventh sheet shows the UNIPROT access of ECM organization signaling pathway proteins from the control group in column A and from the iHDAC group in column B.

Supplemental Table S6 Classification of Extracellular Matrix proteins by the Matrisome System. The first sheet shows the 124 proteins classified in the Matrisome in the Control group. The second sheet shows the 134 proteins classified in the Matrisome in the iHDAC group. The abundance of each protein and a heat map of the variation in abundance between the two treatments are shown on the third and fourth sheets.

Supplemental Table S7 Enrichment analysis of common proteins in the secretome. The first sheet shows total common proteins identified in the secretomes. The second sheet displays the proteins significantly down regulated in iHDAC secretome and the fold change. The third sheet displays the proteins significantly upregulated in iHDAC secretome and the fold change. *P* value < 0,005.

Supplemental Table S8 Dataset comprising an array-based molecular profiling of 1,122 patients newly diagnosed with low grade or high-grade glioma published by Ceccarelli and collaborators 2016. cBioPortal was searched using the proteins found in the secretomes and only proteins whose genes were mutated were analyzed. The table shows the main epidemiological, clinical, pathophysiological features and genomic alterations related to 43 patients. Green cells highlight genomic alterations associated with grade 2 glioma, yellow cells highlight genomic alterations associated with grade 3 glioma and red cells highlight genomic alterations associated with grade 4 glioma, GBM. COSMIC data base was used to map Matrisome proteins mutations in different types of cancers and Markov models (FATHMM), through the Analyze Cancer-Associated Variants tool, was used to investigate missense pathogenic mutations with a prediction threshold = −0,75.

Supplemental Table S9 UNIPROT classification of Matrisome proteins related to angiogenesis in each experimental group.
