# Supplementary material for: Epigenetic Mechanisms Histone Deacetylase–Dependent Regulate the Glioblastoma Angiogenic Matrisome and Disrupt Endothelial Cell Behavior In Vitro
Source: Mol Cell Proteomics. 2024 Jan 23;23(3):100722. doi: 10.1016/j.mcpro.2024.100722 (PMC10883839; doi:10.1016/j.mcpro.2024.100722)
Supplement: Supplemental Figures [file mmc1.pdf]

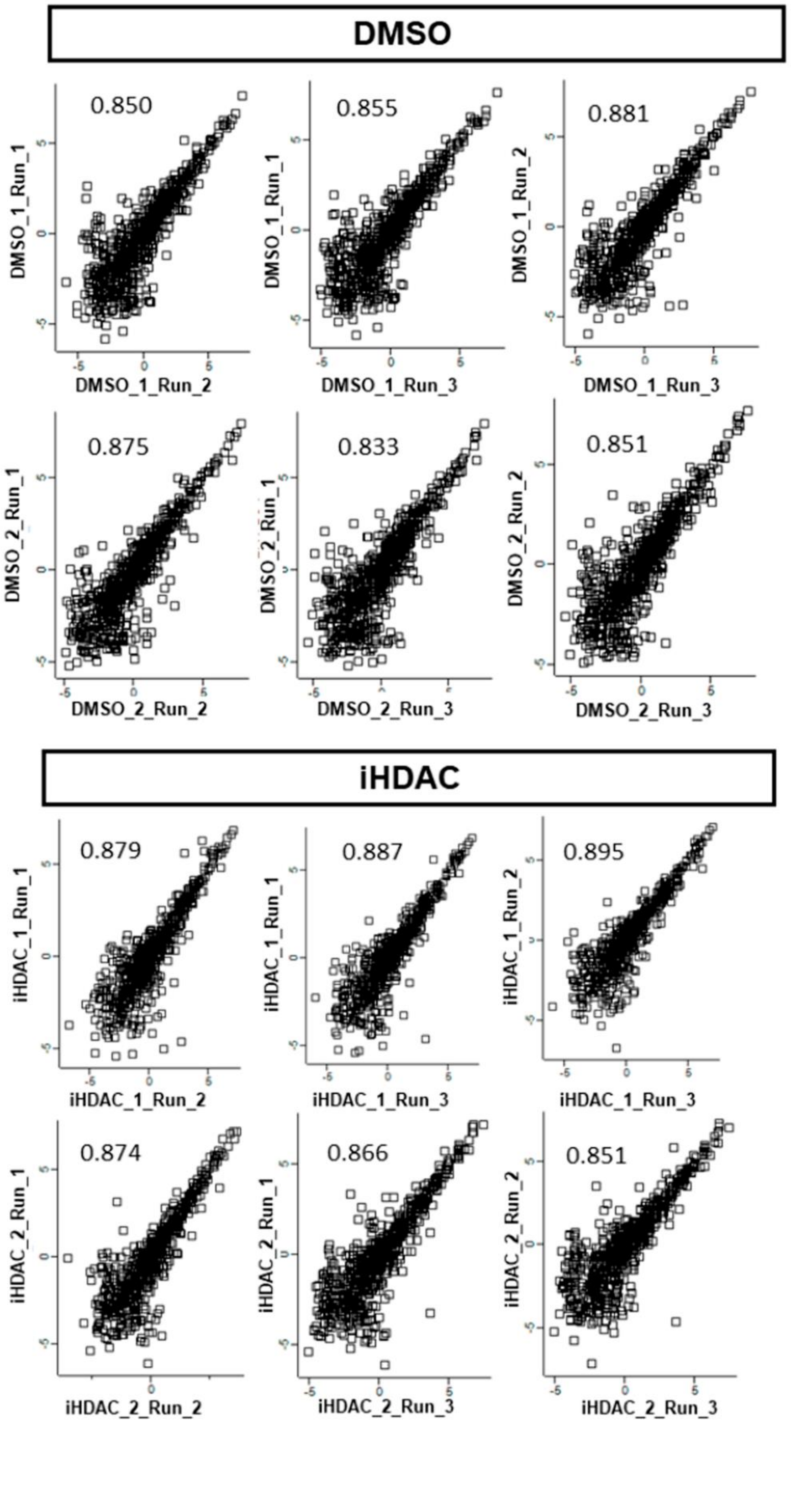

Supplemental Figure 1: Data quality analysis. (a) Pearson correlation of the three experimental replicates runs from each biological sample of DMSO. (b) Pearson correlation of the three experimental replicates runs from each biological sample of TSA.

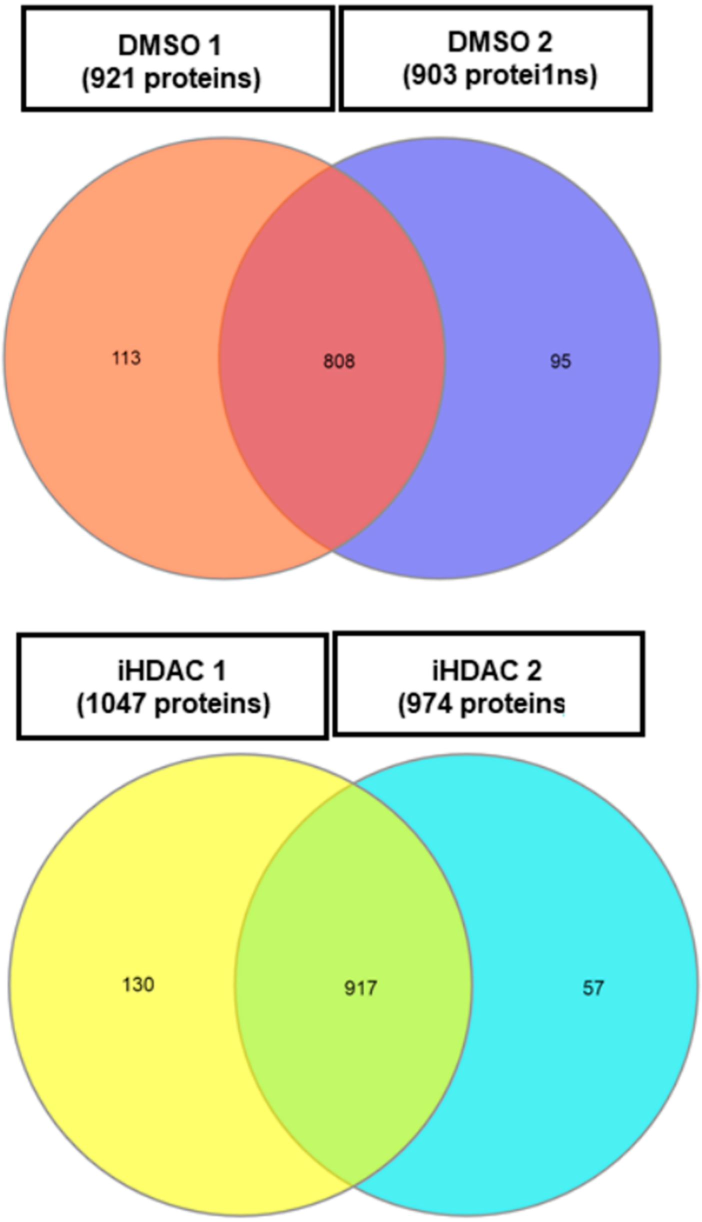

Supplemental Figure 2: Venn Diagram analysis. Venn diagram was used to identify the common proteins identified and quantified in each biological replicate of DMSO1 and DMSO2; and TSA1 and TSA2. For each treatment, the intersection area of the Venn diagram shows the 808 proteins used in the analysis of the DMSO group and the 917 proteins used in the analysis of the iHDAC group.

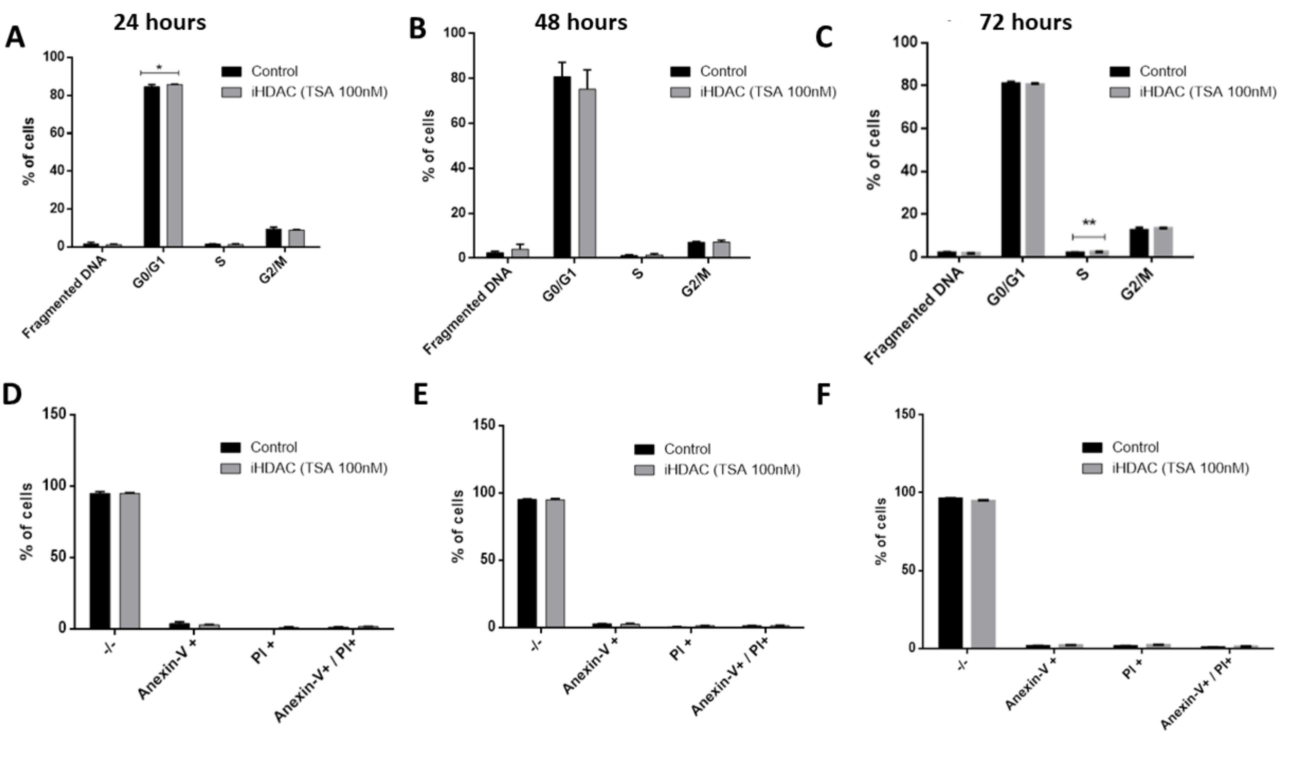

Supplemental Figure 3: Cell cycle progression and survival of U87MG cells at 24, 48 and 72 h was monitored by flow cytometry. (A- C) Graphs represent the percentage of cells in each phase of the cell cycle (G0 – G1, S, G2/ M). (D-F) Cell survival was assessed by Annexin/PI. Figure shows mean of three experiments + SEM. $*p < 0.05$

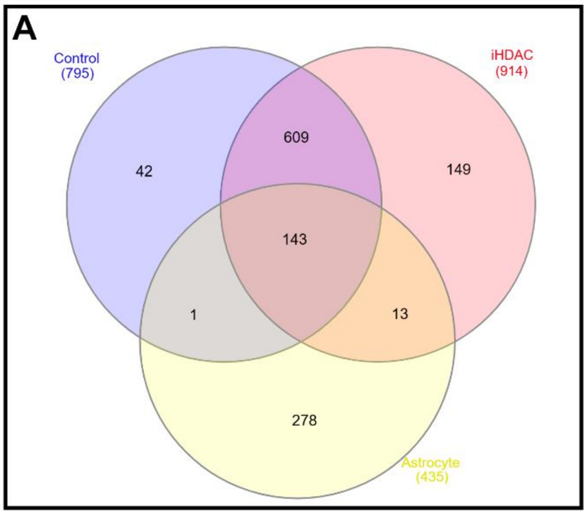

B

Common proteins among GBM and Astrocytes				
UNIPROT ID	Protein	Protein names	Gene Names	Fold change
P08253	MMP2_HUMAN	72 kDa type IV collagenase (EC 3.4.24.24) (72 kDa gelatinase) (Gelatinase A) (Matrix metalloproteinase-2) (MMP-2) (TBE-1) [Cleaved into: PEX]	MMP2 CLG4A	
Q02952	AKA12_HUMAN	A-kinase anchor protein 12 (AKAP-12) (A-kinase anchor protein 250 kDa) (AKAP 250) (Gravin) (Myasthenia gravis autoantigen)	AKAP12 AKAP250	2,7
P01023	A2MG_HUMAN	Alpha-2-macroglobulin (Alpha-2-M) (C3 and PZP-like alpha-2-macroglobulin domain-containing protein 5)	A2M CPAMD5 FWP007	
O75882	ATRN_HUMAN	Attractin (DPPT-L) (Mahogany homolog)	ATRN KIAA0548 MGCA	
Q14019	COTL1_HUMAN	Coactosin-like protein	COTL1 CLP	2
Q9NZV1	CRIM1_HUMAN	Cysteine-rich motor neuron 1 protein (CRIM-1) (Cysteine-rich repeat-containing protein S52) [Cleaved into: Processed cysteine-rich motor neuron 1 protein]	CRIM1 S52 UNQ1886/PRO4330	
P07585	PGS2_HUMAN	Decorin (Bone proteoglycan II) (PG-S2) (PG40)	DCN SLRR1B	0,4
Q8IXL6	FA20C_HUMAN	Extracellular serine/threonine protein kinase FAM20C (EC 2.7.11.1) (Dentin matrix protein 4) (DMP-4) (Golgi casein kinase) (Golgi-enriched fraction casein kinase) (GEF-CK)	FAM20C DMP4	
Q16658	FSCN1_HUMAN	Fascin (55 kDa actin-bundling protein) (Singed-like protein) (p55)	FSCN1 FAN1 HSN SNL	2,6
P35052	GPC1_HUMAN	Glypican-1 [Cleaved into: Secreted glypican-1]	GPC1	0,45
Q14393	GAS6_HUMAN	Growth arrest-specific protein 6 (GAS-6) (AXL receptor tyrosine kinase ligand)	GAS6 AXLLG	
P34932	HSP74_HUMAN	Heat shock 70 kDa protein 4 (HSP70RY) (Heat shock 70-related protein APG-2)	HSPA4 APG2	2
P22626	ROA2_HUMAN	Heterogeneous nuclear ribonucleoproteins A2/B1 (hnRNP A2/B1)	HNRNPA2B1 HNRPA2B1	0,41
P16402	H13_HUMAN	Histone H1.3 (Histone H1c) (Histone H1s-2)	H1-3 H1F3 HIST1H1D	
P16401	H15_HUMAN	Histone H1.5 (Histone H1a) (Histone H1b) (Histone H1s-3)	H1-5 H1F5 HIST1H1B	
Q16270	IBP7_HUMAN	Insulin-like growth factor-binding protein 7 (IBP-7) (IGF-binding protein 7) (IGFBP-7) (IGFBP-rP1) (MAC25 protein) (PGI2-stimulating factor) (Prostacyclin-stimulating factor) (Tumor-derived adhesion factor) (TAF)	IGFBP7 MAC25 PSF	
P05362	ICAM1_HUMAN	Intercellular adhesion molecule 1 (ICAM-1) (Major group rhinovirus receptor) (CD antigen CD54)	ICAM1	
P35527	K1C9_HUMAN	Keratin, type I cytoskeletal 9 (Cytokeratin-9) (CK-9) (Keratin-9) (K9)	KRT9	
Q14767	LTBP2_HUMAN	Latent-transforming growth factor beta-binding protein 2 (LTBP-2)	LTBP2 C14orf141 LTBP3	2,45
P40925	MDHC_HUMAN	Malate dehydrogenase, cytoplasmic (EC 1.1.1.37) (Aromatic alpha-keto acid reductase) (KAR) (EC 1.1.1.96) (Cytosolic malate dehydrogenase)	MDH1 MDHA	2,8
P55001	MFAP2_HUMAN	Microfibrillar-associated protein 2 (MFAP-2) (Microfibril-associated glycoprotein 1) (MAGP) (MAGP-1)	MFAP2 MAGP1	0,28
P58546	MTPN_HUMAN	Myotrophin (Protein V-1)	MTPN	2,7
Q9H1E3	NUCKS_HUMAN	Nuclear ubiquitous casein and cyclin-dependent kinase substrate 1 (P1)	NUCKS1 NUCKS JC7	
Q14247	SRC8_HUMAN	Src substrate cortactin (Amplaxin) (Oncogene EMS1)	CTTN EMS1	0,33
P78371	TCPB_HUMAN	T-complex protein 1 subunit beta (TCP-1-beta) (CCT-beta)	CCT2 99D8.1 CCTB	
Q15582	BGH3_HUMAN	Transforming growth factor-beta-induced protein ig-h3 (Beta ig-h3) (Kerato-epithelin) (RGD-containing collagen-associated protein) (RGD-CAP)	TGFBI BIGH3	

Legend:
Common proteins GBMXAstrocyte (no fold change)
Up regulated in iHDAC
Down regulated in iHDAC
Protein shared between GBM_Control and Astrocyte
Protein shared between GBM_iHDAC and Astrocyte

Suppl. Figure 4 Analysis of the normal human astrocyte secretome according to Kim et al 2022: (A) A set of 143 proteins in the normal human secretome was found to be common with GBM control and iHDA secretomes, 1 protein protein was common to the control secretome and 13 proteins were common to the iHDAC secretome (B) The set of 26 proteins found in the normal human astrocyte secretome whose fold change was significantly downregulated (blue) or up regulated (red). The protein detected only in the control secretome is indicated in yellow and the 13 proteins shared with the iHDAC secretome are indicated in purple.

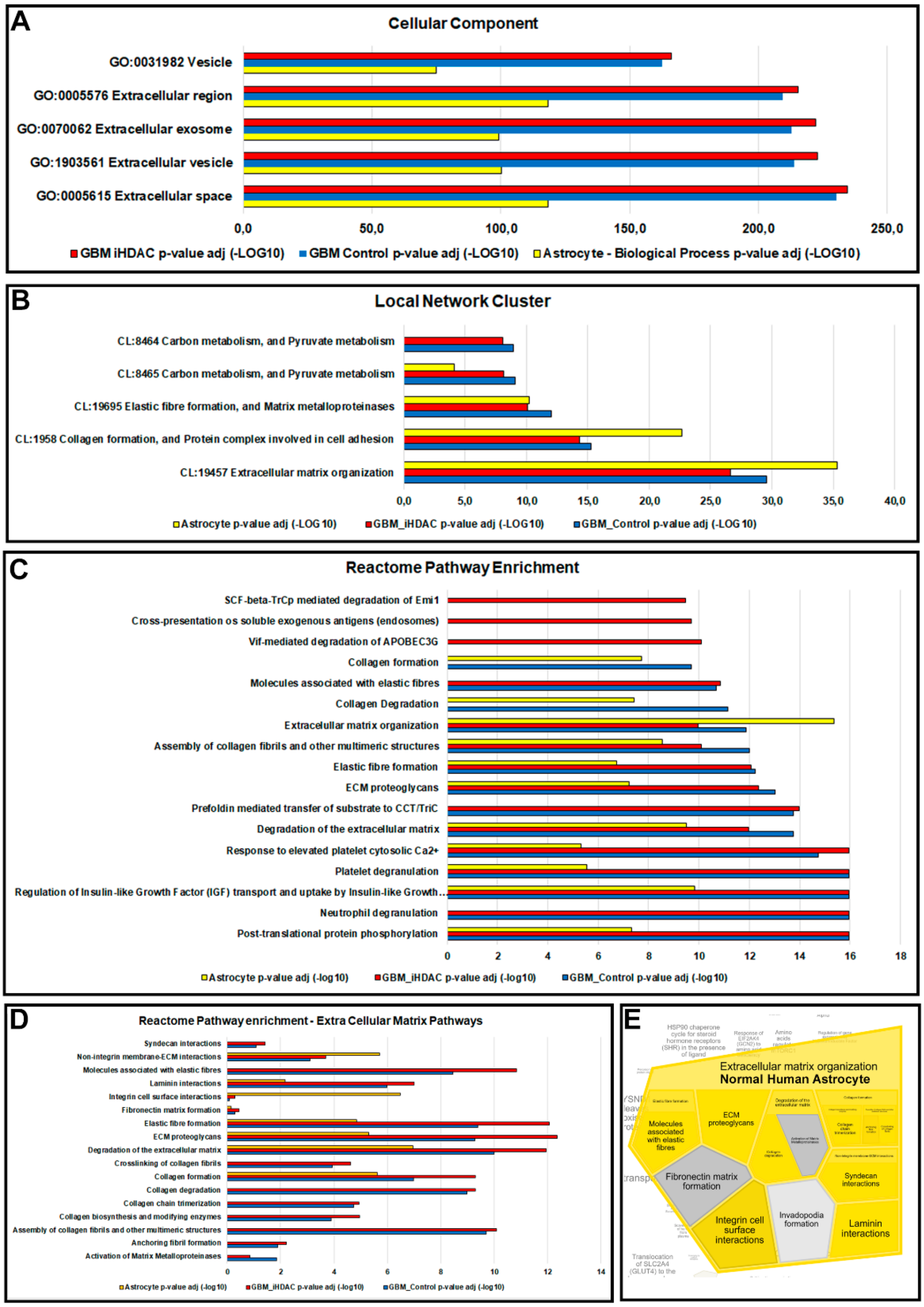

Suppl. Figure 5 The top 5 cellular component (A) and local network cluster (B) were selected and ranked according to the FDR *P* value showing the most enriched components in the secretome of normal human astrocyte (yellow bar) in comparison to control (blue bar) or to iHDAC (red bar) secretomes. (C) REACTOME pathway enrichment analysis of the normal human astrocyte (yellow bar) illustrating the 17 statistically significant pathways ($p<0.05$) characterized in the GBM control (blur bar) and iHDAC (red bar) secretomes are listed and the p values for pathway enrichment are adjusted to -log10 (padj) (D) Enrichment analysis of Extracellular Matrix Organization sub pathways found in Reacfoam that are significantly enriched ($p<0.05$; p value adjusted to -log10 (padj) in normal human astrocytes (yellow bar), control (blur bar) or iHDAC (red bar) secretomes. (E) Reacfoam or Voronoi graph depicts only the components of the normal human astrocyte secretome that belong to the Extracellular Matrix Organization according to REACTOME classification. Each polygon represents Extracellular Matrix Organization sub pathways and the area of each polygon is directly related to the number of proteins identified in the secretome that map in each sub pathway.

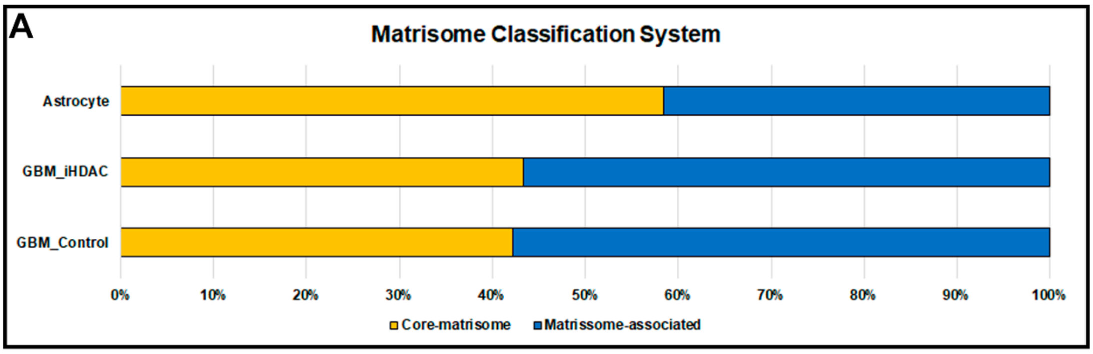

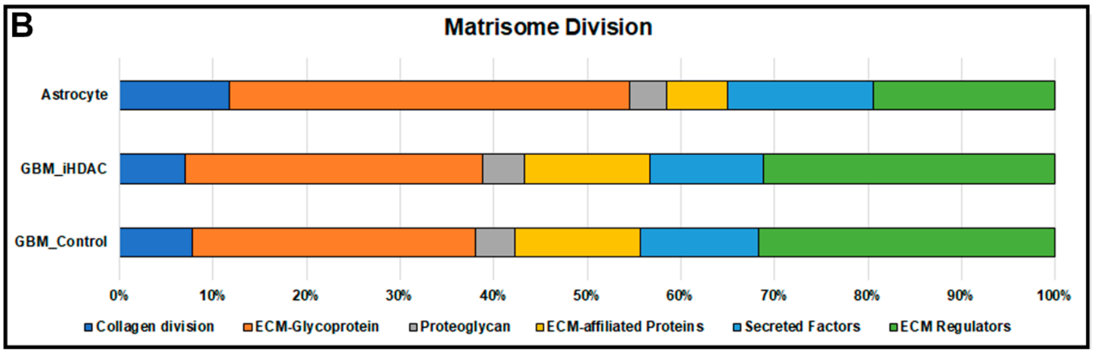

C

Matrisome common proteins GBMXAstrocyte

UNIPROT ID	Protein	Protein names	Annotated gene	Matrisome category	Matrisome division	Proteins in each group	Fold change
P07585	PGS2_HUMAN	Decorin (Bone proteoglycan II) (PG-S2) (PG40)	DCN SLRR1B	Core matrisome	Proteoglycans		0,4
Q9NZV1	CRIM1_HUMAN	Cysteine-rich motor neuron 1 protein (CRIM-1) (Cysteine-rich repeat-containing protein S52) [Cleaved into: Processed cysteine-rich motor neuron 1 protein]	CRIM1 S52 UNQ1886/PRO4330	Core matrisome	ECM Glycoproteins		
Q14393	GAS6_HUMAN	Growth arrest-specific protein 6 (GAS-6) (AXL receptor tyrosine kinase ligand)	GAS6 AXLLG	Core matrisome	ECM Glycoproteins		
Q14767	LTBP2_HUMAN	Latent-transforming growth factor beta-binding protein 2 (LTBP-2)	LTBP2 C14orf141 LTBP3	Core matrisome	ECM Glycoproteins		2,45
P55001	MFAP2_HUMAN	Microfibrillar-associated protein 2 (MFAP-2) (Microfibril-associated glycoprotein 1) (MAGP) (MAGP-1)	MFAP2 MAGP1	Core matrisome	ECM Glycoproteins		0,28
P01008	ANT3_HUMAN	Antithrombin-III (ATIII) (Serpin C1)	SERPINC1 AT3 PRO0309	Matrisome-associated	ECM Regulators		
Q8IXL6	FA20C_HUMAN	Extracellular serine/threonine protein kinase FAM20C (EC 2.7.11.1) (Dentin matrix protein 4) (DMP-4) (Golgi casein kinase) (Golgi-enriched fraction casein kinase) (GEF-CK)	FAM20C DMP4	Matrisome-associated	ECM Regulators		
P09603	CSF1_HUMAN	Macrophage colony-stimulating factor 1 (CSF-1) (M-CSF) (MCSF) (Lanimostim) (Proteoglycan macrophage colony-stimulating factor) (PG-M-CSF) [Cleaved into: Processed macrophage colony-stimulating factor 1; Macrophage colony-stimulating factor 1 43 kDa subunit]	CSF1	Matrisome-associated	Secreted Factors		0,47
P35052	GPC1_HUMAN	Glypican-1 [Cleaved into: Secreted glypican-1]	GPC1	Matrisome-associated	ECM-affiliated Proteins		0,45

Legend:
Detected only in iHDAC
Down regulated iHDAC
Up regulated iHDAC

Suppl. Figure 6: Matrisome signature of normal human astrocytes. Matrisome classification of proteins detected in the control and iHDAC secretomes. Each protein detected in the secretomes was classified using the Matrisome classification system (A) and Matrisome division (B). Proteins were sorted according to collagen division, ECM-glycoprotein, proteoglycan, ECM-affiliated protein, secreted factor or ECM regulator and their overlap with GBM experimental groups (C).

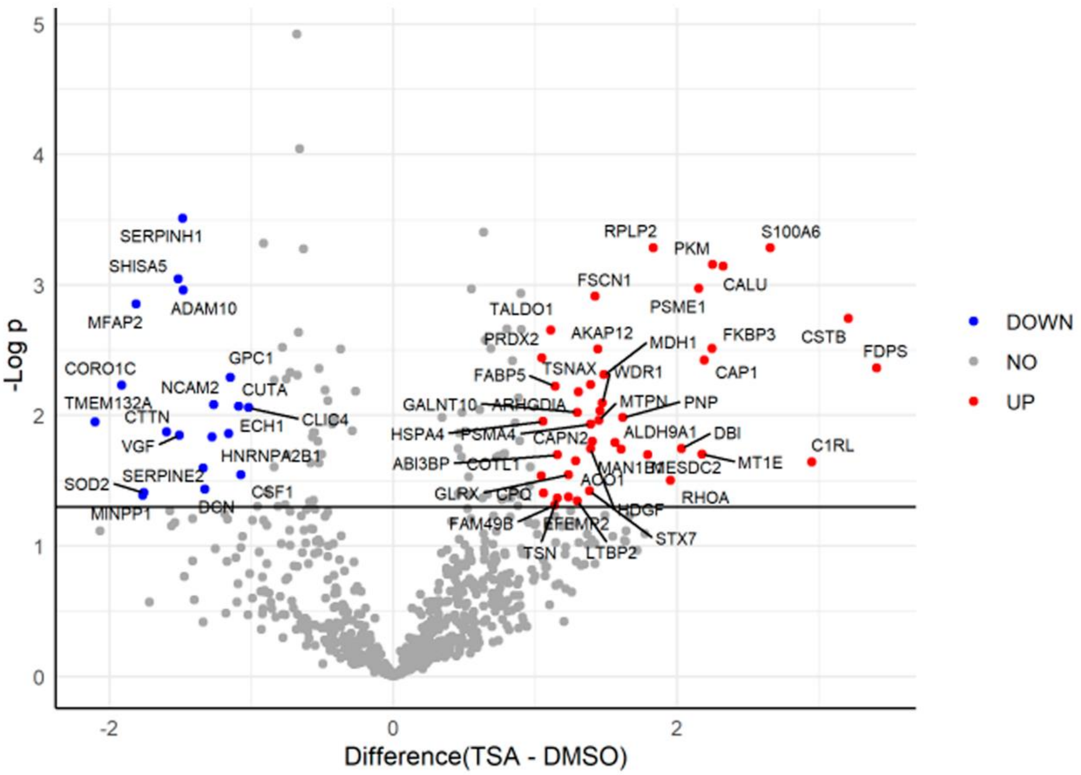

Suppl. Figure 7 Volcano Plot of differentially regulated proteins ($p<0.05$) in response to treatment with iHDAC (red/up-regulated; blue/down-regulated). Proteins were plotted by difference (TSA-DMSO) and significance (-Log p) using a False Discovery Rate (FDR) of 0.05 and $p=0.05$.

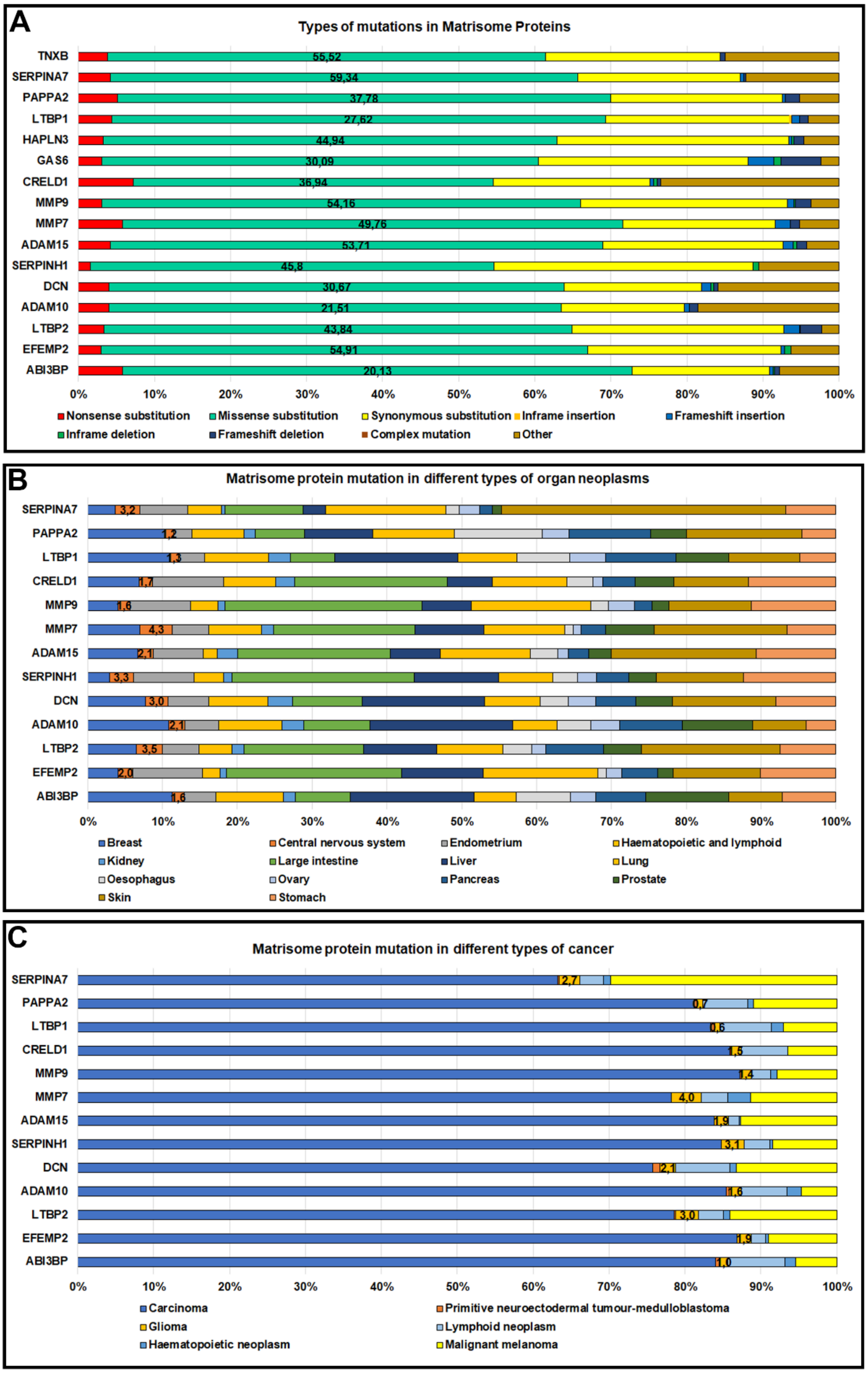

Suppl. Figure 8. Analysis of Matrisome proteins mutation according to the mutation type (A), organ specific neoplasms (B) or type of cancer (C). COSMIC data base was used to screen clinical data for Matrisome proteins harboring mutations.

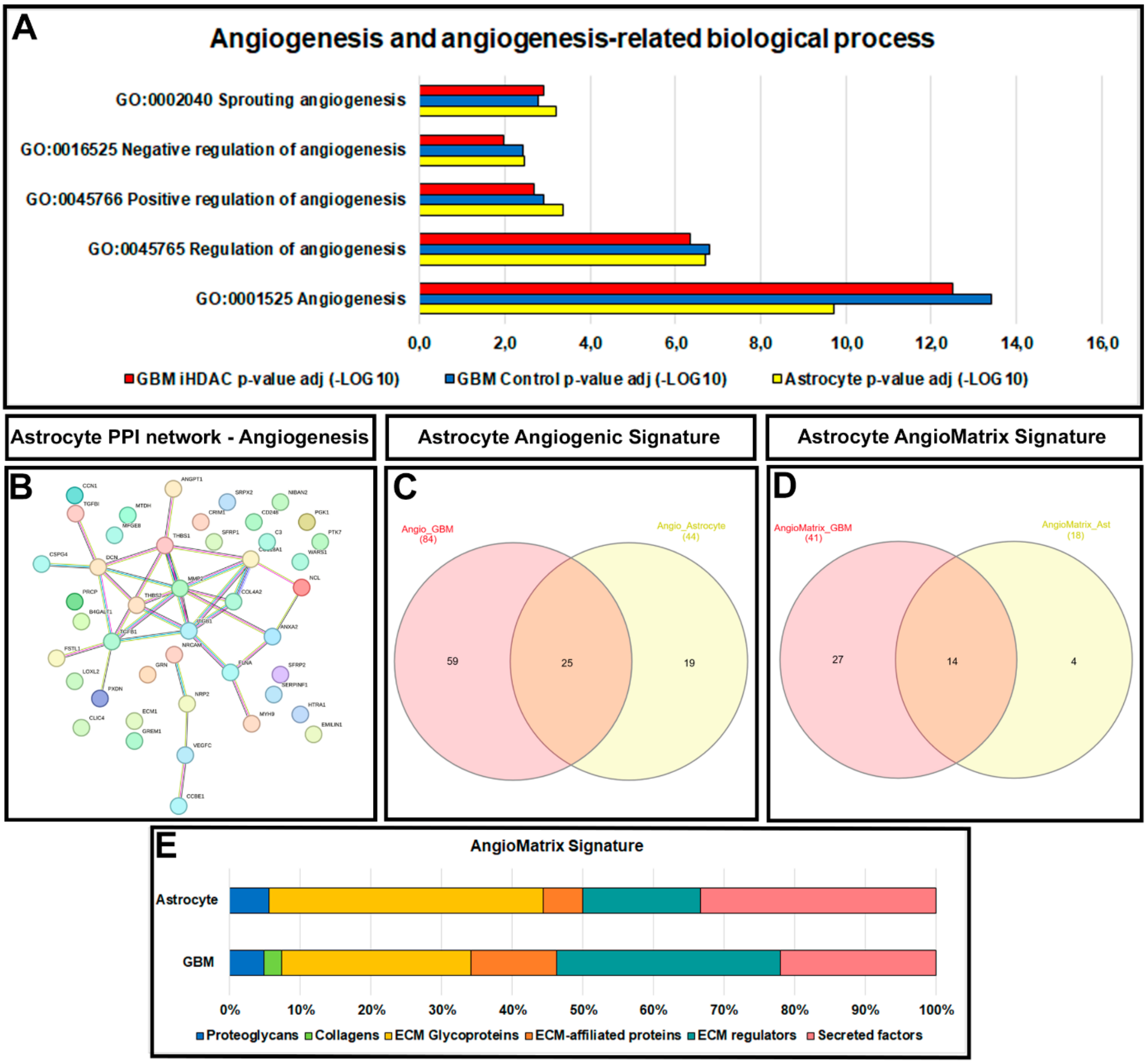

F

UNIPROT ID	Astrocyte AngioMatrix Signature	Protein in each group	Matrisome Division	Matrisome Category
P07585	Decorin (Bone proteoglycan II) (PG-S2) (PG40)	0,4	Core matrisome	Proteoglycans
O60687	Sushi repeat-containing protein SRPX2 (Sushi-repeat protein upregulated in leukemia)		Core matrisome	ECM Glycoproteins
P07996	Thrombospondin-1 (Glycoprotein G)		Core matrisome	ECM Glycoproteins
P35442	Thrombospondin-2		Core matrisome	ECM Glycoproteins
Q08431	Lactadherin (Breast epithelial antigen BA46) (HMFG) (MFGM) (Milk fat globule-EGF factor 8) (MFG-E8) (SED1) [Cleaved into: Lactadherin short form; Medin]		Core matrisome	ECM Glycoproteins
Q16610	Extracellular matrix protein 1 (Secretory component p85)		Core matrisome	ECM Glycoproteins
Q92626	Peroxidasin homolog (EC 1.11.2.-) (Melanoma-associated antigen MG50) (Peroxidasin 1) (hsPxd01) (Vascular peroxidase 1) (p53-responsive gene 2 protein) [Cleaved into: PXDN active fragment]		Core matrisome	ECM Glycoproteins
Q9NZV1	Cysteine-rich motor neuron 1 protein (CRIM-1) (Cysteine-rich repeat-containing protein S52) [Cleaved into: Processed cysteine-rich motor neuron 1 protein]		Core matrisome	ECM Glycoproteins
Q6UVK1	Chondroitin sulfate proteoglycan 4 (Chondroitin sulfate proteoglycan NG2) (Melanoma chondroitin sulfate proteoglycan) (Melanoma-associated chondroitin sulfate proteoglycan)		Matrisome-associated	ECM-affiliated Proteins
P08253	72 kDa type IV collagenase (EC 3.4.24.24) (72 kDa gelatinase) (Gelatinase A) (Matrix metalloproteinase-2) (MMP-2) (TBE-1) [Cleaved into: PEX]		Matrisome-associated	ECM Regulators
Q92743	Serine protease HTRA1 (EC 3.4.21.-) (High-temperature requirement A serine peptidase 1) (L56) (Serine protease 11)		Matrisome-associated	ECM Regulators
Q9Y4K0	Lysyl oxidase homolog 2 (EC 1.4.3.13) (Lysyl oxidase-like protein 2) (Lysyl oxidase-related protein 2) (Lysyl oxidase-related protein WS9-14)		Matrisome-associated	ECM Regulators
P01137	Transforming growth factor beta-1 proprotein [Cleaved into: Latency-associated peptide (LAP); Transforming growth factor beta-1 (TGF-beta-1)]		Matrisome-associated	Secreted Factors
P49767	Vascular endothelial growth factor C (VEGF-C) (Flt4 ligand) (Flt4-L) (Vascular endothelial growth factor-related protein) (VRP)		Matrisome-associated	Secreted Factors
Q12841	Follistatin-related protein 1 (Follistatin-like protein 1)		Matrisome-associated	Secreted Factors
Q15389	Angiopoietin-1 (ANG-1)		Matrisome-associated	Secreted Factors
Q6UXH8	Collagen and calcium-binding EGF domain-containing protein 1 (Full of fluid protein homolog)		Matrisome-associated	Secreted Factors
Q8N474	Secreted frizzled-related protein 1 (FRP-1) (sFRP-1) (Secreted apoptosis-related protein 2) (SARP-2)		Matrisome-associated	Secreted Factors

Legend:
Common with GBM
Down regulated iHDAC
Only in Astrocyte
Only in iHDAC

Suppl. Figure 9 Characterization of the normal human astrocyte AngioMatrix signature. UNIPROT classification of proteins related to angiogenesis (A,B,C) was merged with the Matrisome classified proteins giving rise to the 14 proteins that characterized the normal human astrocyte AngioMatrix signature. (E) Comparison of the AngioMatrix signature of normal and transformed astrocytes. (F) Analysis of the normal human astrocyte AngioMatrix signature according to their overlap with experimental group.
